# The novel *E. coli* cell division protein, YtfB, plays a role in eukaryotic cell adhesion

**DOI:** 10.1038/s41598-020-63729-7

**Published:** 2020-04-21

**Authors:** Amy L. Bottomley, Elizabeth Peterson, Gregory Iosifidis, Adeline Mei Hui Yong, Lauren E. Hartley-Tassell, Shirin Ansari, Chris McKenzie, Catherine Burke, Iain G. Duggin, Kimberly A. Kline, Elizabeth J. Harry

**Affiliations:** 1 ithree institute, University of Technology Sydney, 15 Broadway, Ultimo, NSW 2007 Sydney, Australia; 2 Singapore Centre for Environmental Life Sciences Engineering (SCELSE) and the School of Biological Sciences, Nanyang Technological University, 60 Nanyang Drive, Singapore, 637551 Singapore; 3 Institute for Glycomics, Griffith University, Gold Coast, QLD 4222 Mount Gravatt, Australia; 4 School of Life Sciences, University of Technology Sydney, 15 Broadway, Ultimo, NSW 2007 Sydney, Australia

**Keywords:** Bacterial adhesion, Bacteriology

## Abstract

Characterisation of protein function based solely on homology searches may overlook functions under specific environmental conditions, or the possibility of a protein having multiple roles. In this study we investigated the role of YtfB, a protein originally identified in a genome-wide screen to cause inhibition of cell division, and has demonstrated to localise to the *Escherichia coli* division site with some degree of glycan specificity. Interestingly, YtfB also shows homology to the virulence factor OapA from *Haemophilus influenzae*, which is important for adherence to epithelial cells, indicating the potential of additional function(s) for YtfB. Here we show that *E. coli *YtfB binds to N’acetylglucosamine and mannobiose glycans with high affinity. The loss of *ytfB* results in a reduction in the ability of the uropathogenic *E. coli* strain UTI89 to adhere to human kidney cells, but not to bladder cells, suggesting a specific role in the initial adherence stage of ascending urinary tract infections. Taken together, our results suggest a role for YtfB in adhesion to specific eukaryotic cells, which may be additional, or complementary, to its role in cell division. This study highlights the importance of understanding the possible multiple functions of proteins based on homology, which may be specific to different environmental conditions.

## Introduction

The elucidation of bacterial protein function is critical for a fundamental understanding of almost all biological processes. However, the role of a protein may only be discovered under certain conditions such as growth in particular carbon sources, different temperatures, or in a specific environmental niche e.g. during infection of the host. To further complicate our understanding of protein function many proteins have overlapping or redundant roles, making the understanding of a single protein in the context of a whole cell difficult. Added to this is the phenomenon of ‘moonlighting‘proteins, where a single protein performs multiple physiologically relevant functions^1–5^.

We have entered an era where the discovery rate of new genes is rapidly increasing due to the high throughput capability of Next Generation Sequencing. However, characterisation of these gene products (proteins) still lags behind. Additionally, the use of genome-wide screens, including transposon mutant libraries, TRADIS or whole-genome shotgun library screens, has implicated many genes as being important under a specific environmental condition, but the mechanistic role of the gene product is still poorly understood. Not surprisingly, it has been reported that ~24% of proteins have no functional prediction from a database of completely sequenced microbial genomes. A large proportion of proteins (76%) have a predicted function based on computational functional homology assignmentbut less than 1% of these proteins have been experimentally characterised^6^. Whilst computational prediction can be powerful, it may result in inaccurate assignment of function based solely on homology searches^7^ and does not accommodate the possibility of protein multifunctionality. Therefore, it is necessary to experimentally confirm predicted protein function using homology alignments as a guide, but also taking into consideration the possibility of complementary or additional functions depending on the environmental condition.

**Figure 1:**
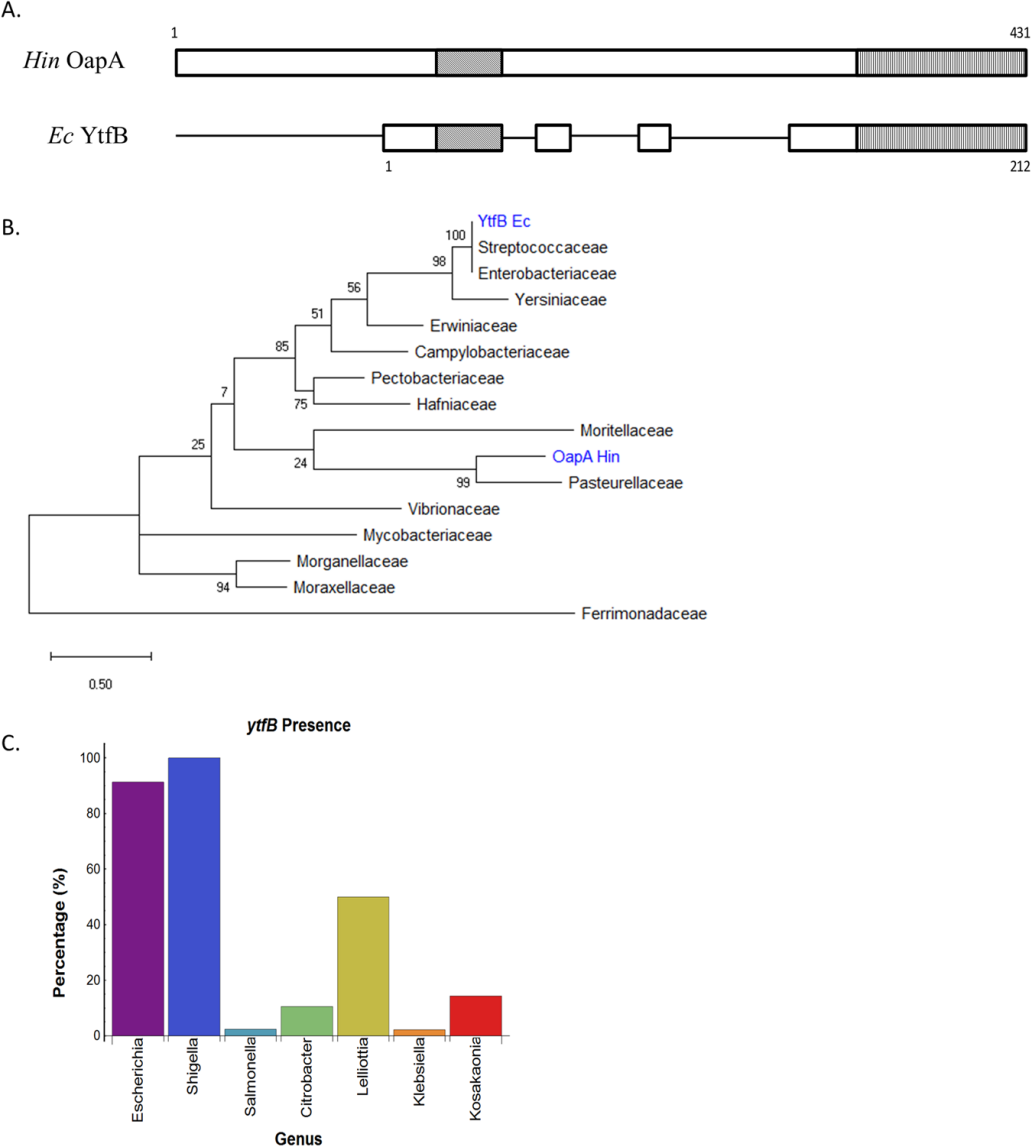
*E. coli* YtfB shows homology to *H. influenzae* OapA, and is conserved in *Enterobacteriaceae*. (**A**) Schematic of *H. influenzae *(Hin) OapA and *E. coli* (Ec) YtfB proteins. Solid boxes show areas of homology, as predicted by Clustal Omega.Two homologous domains are predicted: the OapA domain (diagonal lines) and the LysM-like domain (vertical lines). (**B**) Phylogenetic tree of YtfB homologues from diverse bacterial families.YtfB is primarily found in closely-related Gamma-proteobacteria. *H. influenzae* (Hin) OapA and *E. coli* (Ec) YtfB are highlighted in blue. (**C**) YtfB is most highly conserved in *Enterobacteriaceae*. Frequency of conservation of YtfB amongst genera within this family was calculated by dividing the number of strains containing YtfB by the total number of strains available in the database.

Previously, we performed a genome-wide screen to identify novel cell division genes and regulators^8^. From this screen, 12 distinct loci were identified, 7 of which contained at least one ‘*y*’ gene of unknown function^8^. We became interested in *ytfB*, because high expression levels caused cell division inhibition in *Escherichia coli*, resulting in filamentous cells^8^. Very recently, a role for YtfB in cell division was reported, with the protein initially selected for investigation due to computational annotation as containing a peptidoglycan binding domain^9^. Interestingly, bioinformatic analysis showed that YtfB shares homology with the virulence factor OapA in *Haemophilus influenzae* across 2 domains: a short sequence spanning the transmembrane domain proximal to the N-terminus; and a LysM-like domain at the extreme C-terminus^9^ (Fig. 1A). This suggests, based on protein homology predictions, there may be a function(s) of YtfB in addition to its role in cell division. LysM domains are involved in binding to polysaccharides found on bacterial, plant and eukaryotic cell surfaces, and are present in proteins that have a variety of functions, including adherence and virulence^10^. Indeed, *H. influenzae *OapA expression is critical for the initial adherence to rat nasal epithelial cells during upper respiratory infections^11^ and the extracellular domain binds directly to Chang epithelial cells^12^. YtfB has also been shown to bind to denuded glycan (i.e. glycans with stem peptides removed) present in the bacterial cell wall^9^, suggesting a possible role in glycan binding for YtfB.

Here, we show that the cell division protein YtfB, which shows homology to the adhesin OapA necessary for *H. influenzae* virulence, binds with high affinity to N’acetylglucosamine and mannobiose glycans. We further investigated the function of YtfB using the uropathogenic *E. coli* strain UTI89 and found that the loss of *ytfB* results in a reduction in the ability UTI89 to adhere to kidney cells, but not to bladder cells, indicating a specific role the initial adherence stage of ascending urinary tract infections. Taken together, our results suggest a role for YtfB in the switch of a motile to a sessile lifestyle in the environment of the urinary tract, which may be additional, or complementary, to its role in cell division.

## Results

### *ytfB* is primarily conserved in Enterobacteriaceae

To investigate if association with certain environments or clades could give insights into the function of YtfB, the phylogenetic conservation of the protein was investigated. The amino acid sequence from *E. coli* MG1655 was used to identify homologues from genetically diverse families using BLAST^13^ and JackHMMER^14^, and a phylogenetic tree was produced using MEGA X^15^. The sequence identity of *E. coli* YtfB to identified homologues ranged between 23% and 100%. Overall, YtfB was found primarily in Gamma-proteobacteria, with single homologues identified in Epsilon-proteobacteria, Firmicutes and Actinobacteria (Fig. 1B). Identification of ancestral sequences indicates that *Pastuerellaceae* (of which *H. influenzae* is a member) is the probable ancestor. The majority of YtfB homologues are present in the *Enterobacteriaceae* family, with *Escherichia* and *Shigella* being the dominant highly conserved species. Further investigation showed that within these species, almost all strains of *Escherichia* and *Shigella* contained aYtfB homologue indicating a high degree of conservation, whilst other species showed a lower conservation of the gene amongst strains (Fig. 1C). Thus, YtfB is primarily conserved within the *Enterobacteriaceae* among species found within the human gastrointestinal tract, although there was no clear correlation between either pathogenic or commensal strains.

Large-scale genome interactome studies of *E. coli* have reported that YtfB interacts with a number of proteins involved in cellular function, as well as the hypothetical fimbrial-like proteins YbgP, YbgD and YgiL^16,17^, whilst analysis of physical and/or functional interactions using STRING^18^ predicts a moderate interaction with the cell division protein DamX, amongst other proteins. It has been observed that YtfB has similar phenotypic characteristics to DamX^9^, and DamX has been further implicated in reversible filamentation during the infective cycle of uropathogenic *E. coli* (UPEC) in a murine model of cystitis^19^. Therefore, based on literature and sequence homology to a known virulence factor in *H. influenzae*, we hypothesised that YtfB could have a role in adhesion, either during commensal symbiosis within a host, or during infection.

### YtfB is associated with the outer membrane

Homology to OapA and the presence of a LysM-like domain suggests that YtfB may be a surface adhesin involved in adherence to glycan moieties. A Kyte Doolittle hydropathy plot predicts a hydrophobic domain between residues 33 and 49, indicative of a membrane-spanning domain. However, the cellular localisation of YtfB is less clear; predictions based on homology to *H. influenzae *OapA suggest that YtfB is associated either with the inner membrane, with the large C-terminal domain facing outwards, or the extracellular space (PSORTb, HMMTOP and TMHMM). Predicted extracellular localisation is unusual as there is no prokaryotic signal sequence in YtfB; however, no signal sequence is computationally predicted for OapA, which has been experimentally confirmed to be present in the inner and outer membranes of *H. influenzae* cells in equal amounts^11^.

**Figure 2:**
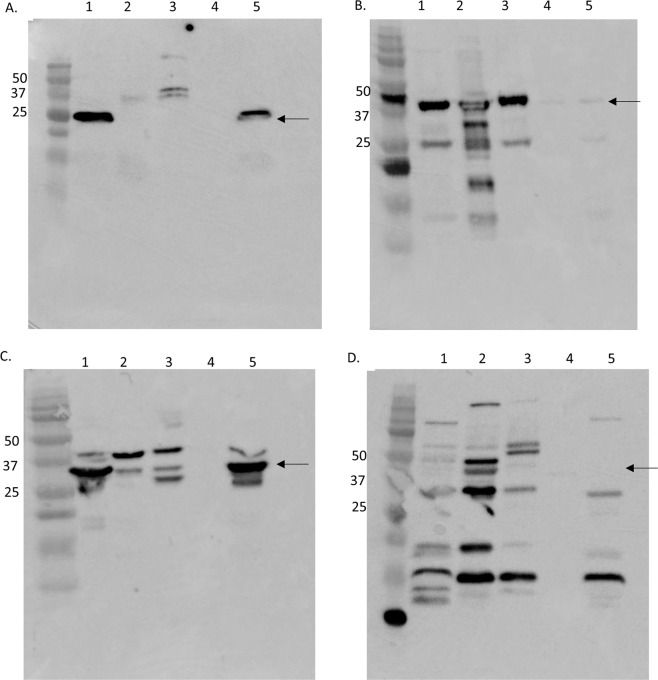
Expression and cellular location of YtfB. BW25113 expressing *ytfB-FLAG* under control of the native promoter was fractionated into its substituent parts and volumes corresponding to equal proportions of the growth culture medium were separated by SDS-PAGE. Western blotting was performed using α-FLAG antibodies, showing YtfB-FLAG associated primarily with the outer membrane (5) fractions. (**A)** As a control for cell fractionation, the blot was also probed with α-FtsZ (**B**), α-OmpA (**C**) and α-maltose binding protein (**D**) antibodies.1 – whole cell lysate; 2 – cytoplasm; 3 – inner membrane; 4 – periplasm; 5 – outer membrane. Sizes of protein standards are highlighted. Arrows indicate the expected size in kDa for each protein: YtfB-FLAG – 23.5 kDa; FtsZ – 40 kDa; OmpA – 35 kDa; MBP 44.9 kDa.

To determine whether YtfB is expressed during exponential growth and to determine its cellular localisation, we constructed a FLAG-fusion of YtfB with expression driven by the native promoter in BW25113. Cells were fractionated into their constituents and probed using an antibody to the FLAG moiety (Fig. 2). A protein of ~25 kDa, corresponding to the predicted size of full-lengthYtfB-FLAG, was detected primarily in the outer membrane fraction. As a control, the cell division protein FtsZ was shown to be distributed mainly in the cytoplasm and less so inner membrane (Fig. 2B), as expected^20^, whilst the outer membrane protein A was largely associated with the outer membrane, as well as the cytoplasm and inner membrane to a lesser degree. Although the maltose binding protein is predicted to be localised with the inner membrane, a large number of bands were detected in several fractions, either due to non-specific detection by the antibody, or degradation of the protein (Fig. 2D). Nevertheless, localisation of *H. influenzae *OapA was previously determined as extracellular using a PhoA reporter gene fusion^11^, which is consistent with the association of YtfB (at least partially) with the outer membrane in *E. coli* (Fig. 2).

### Loss of *ytfB* does not result in observable changes in LPS profile of UTI89

*H. influenzae* colonies can undergo phase variation, with cells from transparent colonies efficiently colonising rat nasopharanges, whilst those isolated from opaque colonies are deficient in colonisation^11^. This phase variation correlates with OapA expression, which is expressed during the transparent phase i.e. during initial host colonisation. Whilst the basis of different colony opaqueness has not been identified, the molecular mass of LPS has been suggested to be larger in transparent colonies (when OapA is expressed) compared to opaque colonies^11^ which may suggest a functional link between LPS size and the role of OapA adhesion to host cells. We did not observe any differences in growth rate, cellular morphology or cell size between BW25113 and the isogenic mutant BW25113Δ*ytfB *(data not shown), ruling out a role for YtfB in the maintenance of gross cellular morphology, in agreement with previous data^9^. We therefore investigated if changes in LPS mass was also observed in the presence and absence of *ytfB*. K-12 strains, including BW25113, do not produce the O antigen repeat unit polysaccharide, which gives rise to LPS of different mass^21^ and we therefore investigated changes in LPS mass in the uropathogenic *E. coli* strain UTI89 which was originally isolated from a patient with cystitis^22^. LPS was extracted from equal biomass of UTI89 and UTI89Δ*ytfB* and the profiles were compared by SDS-PAGE. However, there were no discernible differences in the LPS profile (O antigen, outer core, inner core or lipid A) (Supplementary Fig. 1A) between wild type and mutant cells. Furthermore, using both solid and liquid assays, there was no difference in the bactericidal activity of lysozyme, protection against which is mediated via LPS^23^ (Supplementary Fig. 1B). Together these results suggest that there is no difference in LPS structure in the absence of *ytfB*. There is conflicting evidence about the LPS profile when *H. influenzae* is undergoing phase variation (i.e. transparent or opaque colony formation), with reports of an increase in LPS mass in transparent colonies^11^, as well as no difference in the LPS profile between transparent and opaque colonies (which correlates with presence and absence of OapA expression, respectively) in *H. influenzae*^24^. Whilst the link between LPS size and OapA expression remains unclear in *H. influenzae*, we have found that YtfB function is not mediated through gross changes to cell wall structure.

### Interaction of YtfB with glycans

Homology to the *H. influenzae *adhesin OapA, and the predicted presence of a domain involved in binding to glycans raised the question of whether YtfB directly binds to glycans present on host cell surfaces. The ability of bacteria to bind to host tissue is often paralleled by the ability of the bacteria to bind to yeast cells causing agglutination^25^. The surface of yeast cells are rich in mannosylated glycoproteins, and mannose-sensitive adherence is a hallmark of type 1 fimbrial adherence in *E. coli* during establishment of bladder infection in the urinary tract^25,26^. The deletion of *ytfB* in UTI89 resulted in a 10-fold increase in the number of bacteria needed to agglutinate yeast compared to wild type (Supplementary Fig. 2C), whilst deletion of FimH (the mannose-specific adhesin located on the tip of type 1 fimbriae^27^) showed a complete loss of yeast cell agglutination. Agglutination by UTI89 and UTI89 Δ*ytfB *could be competitively inhibited by addition of D-mannose, indicating specific mannose-dependent adherence. The reduction in yeast binding of UTI89 and the corresponding *ytfB* mutant was not due to gross changes in type 1 fimbriae protein levels (Supplementary Fig. 2), which bind to mannosylated proteins present on bladder epithelia^28^, and raises the possibility that YtfB may play a role in adherence to mannosylated glycoproteins.

**Figure 3:**
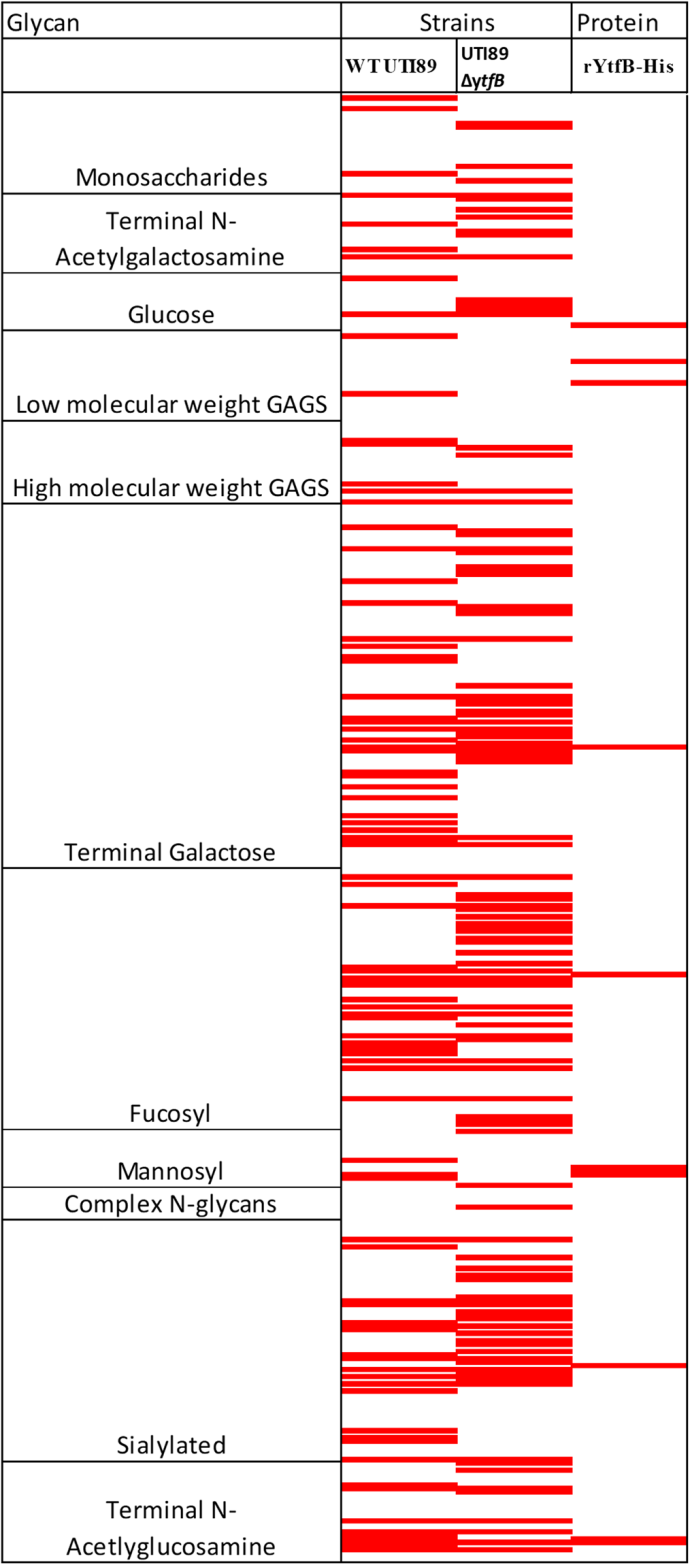
Glycans bound by YtfB in the glycan microarray analysis. The glycan binding properties of wild type (UTI89) are compared to that of the isogenic *ytfB* mutant (UTI89Δ*ytfB*) and purified recombinant YtfB.The code corresponds to the glycan code used in Supplementary Tables 1 and 2. Only the structures that were positive for binding in all three replicates (greater than 1 fold above background and a p value < 0.005) are listed as red, whilst white indicates either no binding observed, or binding was not observed in all three replicates.

To investigate this further, we characterised the glycan binding ability of YtfB using glycan microarray analysis. Initially, purified recombinant His_6_-tagged BW25113 YtfB, corresponding to the C- terminal region following the predicted transmembrane domain (residues 52–212, containing the LysM-like domain), was incubated with a glycan array of 415 distinct glycan structures, and revealed binding to 10 unique moieties (Fig. 3). Binding to N’acetylglucosamine (GlcNAc) structures was anticipated since LysM domains have been well characterised to bind to GlcNAc residues in bacterial peptidoglycan^10^. Additionally, binding to mannobiose structures was observed, which correlates to the mannose-specific reduction in yeast cell agglutination in the absence of *ytfB *(see above). This mannobiose binding was very specific because no binding was observed to the higher mannobiose-3 or mannobiose-5 structures, or the complex N-type glycans. This specificity suggests that the binding pocket of YtfB may only accommodate a disaccharide where additional mannoses may cause steric hindrance or are presented on the array in a way that the protein cannot bind.

To confirm this glycan-binding profile of natively-expressed YtfB, the glycan-binding specificities of UTI89 and UTI89Δ*ytfB* whole cells were also investigated, which provided the most comprehensive glycan binding analysis of UTI89 to date. Interestingly, UTI89Δ*ytfB* cells bound to more glycans (103) compared to UTI89 cells (85), and 39 of these were bound by both strains (Supplementary Table 1). This increase in glycan binding of the UTI89Δ*ytfB* mutant may be because some surface structures that bind glycans are masked by the presence of YtfB, a phenomenon which has also been observed in *Neisseria meningitidis*^29^. There were two specific glycans which only showed binding by both wild type cells and recombinant YtfB protein, but not the UTI89Δ*ytfB* mutant, providing strong evidence of specific binding of these glycans by YtfB. These were 4B (GlcNAcβ1-4GlcNAcβ1-4GlcNAc) and 5E (Manα1-4Man). It should be noted that both the wild type and isogenic mutant showed binding to other N’acetylglucosamine and mannobiose structures, likely due to the variety of cell surface adhesins and proteins which have glycan binding properties. There is only one amino acid difference between the sequence of YtfB from BW25113 and UTI89 (G163D), and so whilst unlikely, it also cannot be ruled out that difference in glycan binding may be due to comparing whole cell binding of UTI89 to recombinant protein binding of BW25113 YtfB. However, the binding to Manα1-4Man was specific to UTI89 and recombinant YtfB only, suggesting a novel function for YtfB in binding to mannose glycans.

### YtfB shows high affinity to N’acetylglucosamine and mannobiose glycans

**Figure 4:**
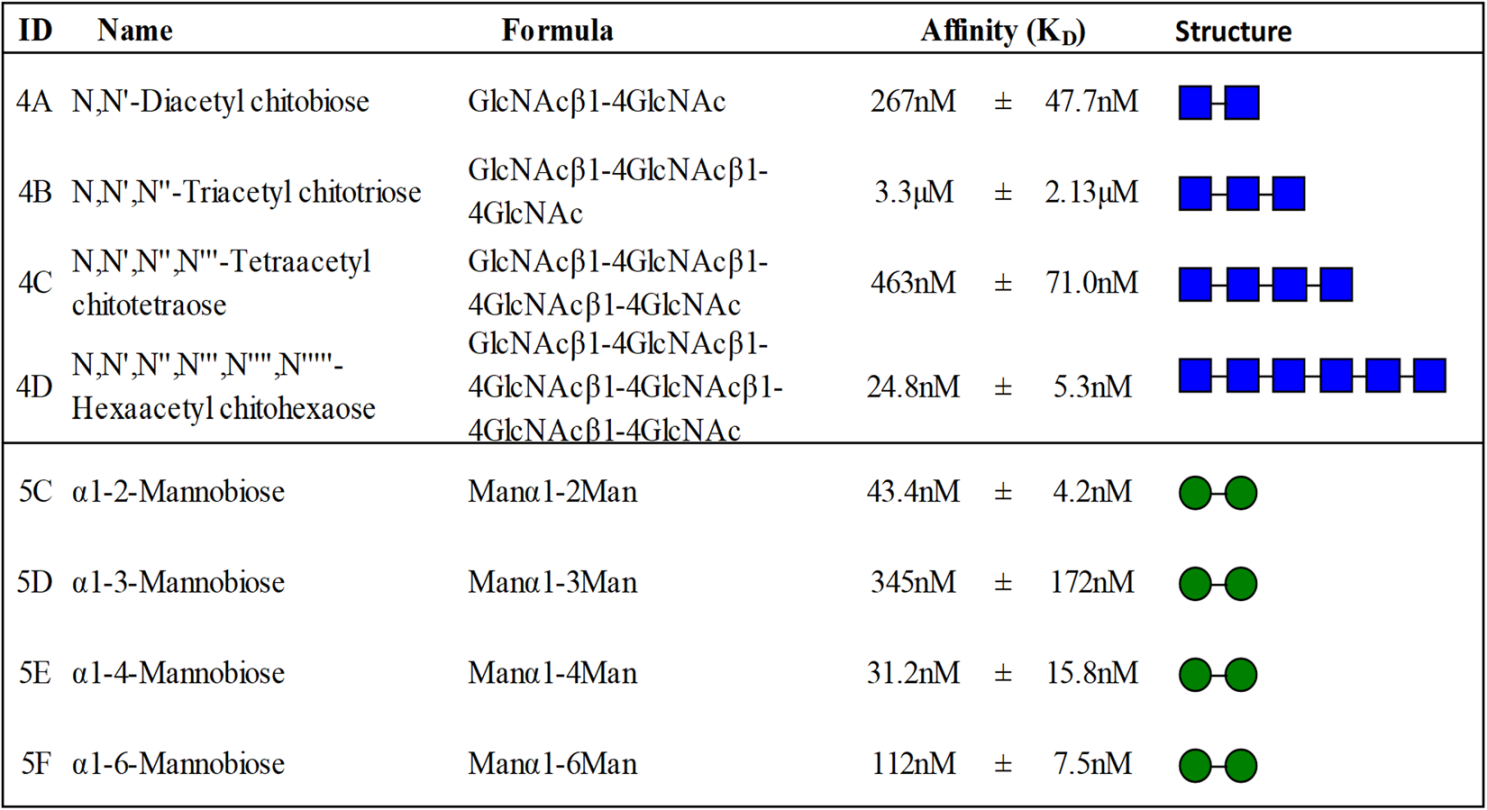
Protein-glycan affinity determination by Surface Plasmon Resonance. Recombinant purified YtfB protein binds to N’acetylglucosamine and mannobiose glycans with various affinities. Affinity constants for the binding interactions were determined using a steady state affinity model and represent three experiments. ID numbers correspond to glycan numbers used in Supplementary Table 2. The structure of glycan moieties are represented by blue squares (GlcNAc) and green circles (Man).

To further characterise the interaction between YtfB and N’acetylglucosamine and mannobiose glycans, the binding affinities were investigated using surface plasmon resonance using the recombinant His-tagged C terminal domain of YtfB as the target protein and a range of glycans with similar structures to those identified with the glycan microarray assay (i.e. similar to 4B and 5E). Steady state affinity surface plasmon resonance revealed out of the four N’acetylglucosamine glycans tested, hexaacetylchitohexaose (4D) showed the highest affinity to YtfB with a KD of 24.8 nM±5.3 nM, as shown in Fig. 4. Binding to this structure was not detected in the glycan microarray with the recombinant YtfB protein, only arrays performed with the whole cells, and may indicate that YtfB requires native folding on the cell surface for binding to a range of N’acetylglucosamine glycans to occur. Of the N’acetylglucosamine structures, only the tetraose showed binding to the *ΔytfB *mutant whole cells. Likewise, only the triose and tetraose showed binding to the recombinant protein. Surface plasmon resonance analysis of the recombinant YtfB to the N’acetylglucosamine structures shows binding to the four repeat lengths.

With the di-mannosylglycans, α1–4mannobiose (5E) had the highest affinity at 31.2 nM, followed by α1–2mannobiose (5 C) with a KD 43.3 nM (Fig. 4). This correlates with the binding observed using the glycan array, where α1–4mannobiose (5E) was bound by YtfB. The other mannobiose structures α1–3mannobiose(5D) and α1–6 mannobiose(5 F) had affinities that were 3–10 fold lower than α1–4mannobiose at 345 nM and 112 nM respectively. α1–2mannobiose (5 C) and α1–3mannobiose (5D) showed binding by recombinant YtfB protein only, whilst α1–6mannobiose (5 F) was only bound by wild type cells; none of these mannose structures were bound by Δ*ytfB* mutant cells, indicating specific binding of di-mannose glycans by YtfB.

In conclusion, using both glycan binding arrays and surface plasmon resonance, we have shown that YtfB binds specifically and with high affinity to N’acetylglucosamine and mannose structures; glycans that are commonly found on host tissue surfaces.

### Deletion of *ytfB* is outcompeted for growth in human urine

**Figure 5:**
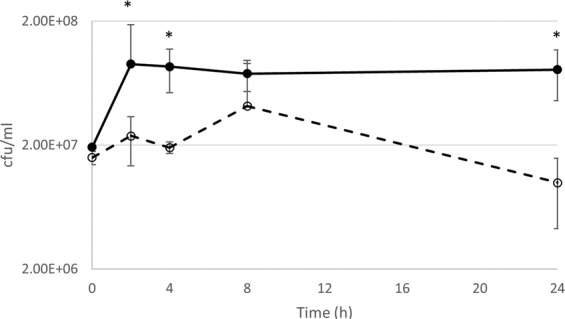
Growth curve of *E. coli* UTI89 and isogenic mutant *ytfB::kan* when grown in competition in human urine. UTI89 (solid line) and UTI89*ytfB::kan* (dashed line) were inoculated to equal starting CFU and growth in human urine was monitored over time, with aliquots being plated at each time point and CFU/ml calculated. The mutant strain showed a reduction in growth up to 8 h, and then showed a drop in CFU whilst UTI89 CFU counts plateaued. The data is an average of three biological replicates; error bars represent SEM. Asterisks indicate P-value < 0.05 as determined by an unpaired student T-test.

Since YtfB binds to mannosylated glycans, which are critical during the initial adhesion of UPEC to bladder cells^28^, we investigated the ability of UTI89Δ*ytfB *to grow in a range of physiologically relevant conditions. Similar to the K-12 non-pathogenic strain (data not shown), no changes in growth rate or cell size were observed in minimal medium, synthetic human urine^30^, human urine or serum (Supplementary Fig. 3). However, growth of UTI89 *ytfB::kan *(using a strain with *ytfB* replaced with a kanamycin resistance cassette to allow for selection) in co-culture with wild type UTI89 in human urine, UTI89 had a higher CFU count than the Δ*ytfB *mutant until 8 h, after which the mutant CFU count decreased whilst wild type CFU plateaued (Fig. 5), indicating that loss of *ytfB* may incur a fitness cost when competing for nutrients in urine in a mixed population.

### Loss of *ytfB* results in a reduction in adherence to human embryonic kidney cells

The impaired ability of the Δ*ytfB *mutant to grow in a co-culture with UTI89, as well as the ability to bind to glycans commonly found on host tissues, suggests a role for *ytfB* in the context of urinary tract infections. We examine the ability of UTI89 and UTI89Δ*ytfB* to adhere to immortalised PD07i human bladder epithelial cells. Compared to the wild type strain there was no significant decrease in adherence with the UTI89Δ*ytfB* mutant. Similarly, there was no difference in the ability of the mutant strain to undergo the intracellular phases of UTI (invasion or intracellular proliferation) compared to wild type (Supplementary Fig. 4).

**Figure 6:**
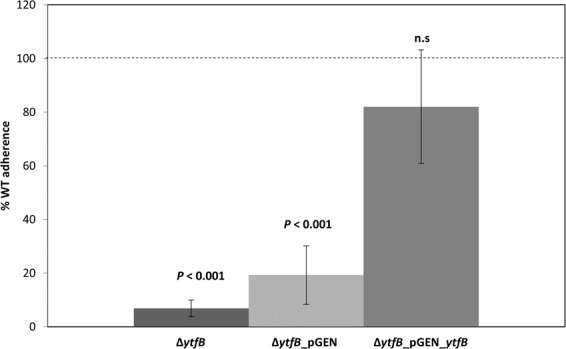
Reduction in adherence to the human kidney cell line HEK-293 when *ytfB* is deleted. Statically grown UTI89, Δ*ytfB*, Δ*ytfB* pGEN and Δ*ytfB* pGEN_ytfB were incubated with a monolayer of HEK-293 cells and adherence measured. The data are averages of eight biological replicates, and error bars represent SEM. Adherence is displayed as a percentage compared to wild type UTI89, whose adherence is represented as a dashed line. P-values were determined using an unpaired student T-test.

During an ascending urinary tract infection, bacteria move from the bladder as the initial site of infection to the kidneys, resulting in pyelonephritis^31^. Thus, the role of YtfB in infection was also investigated, using HEK-293 cells as a model of kidney cell adherence^32^. UTI89Δ*ytfB *cells exhibited significantly reduced adherence (14-fold compared to WT; Fig. 6). Complementation of *ytfB *expression from a plasmid restored adherence to wild type levels (*P* = 0.42). These data suggest that YtfB contributes to the adherence of *E. coli* to human kidney cells, but not to bladder epithelial cells. However, in mouse models of ascending UTI or catheter-associated UTI, UTI89Δ*ytfB* was not attenuated (Supplementary Fig. 5). The absence of an *in vivo* phenotype for UTI89Δ*ytfB* may be due to redundancy of adhesion factors utilised by UTI89 during infection *in vivo*, or may indicate the glycan profile of murine epithelia differs to that of human cells.

### Motility is affected by the deletion of *ytfB*

**Figure 7:**
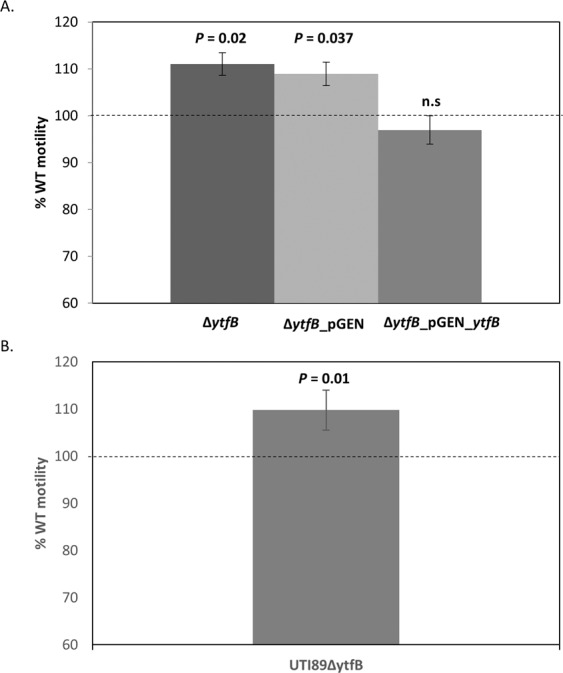
Deletion of *ytfB* results in increased motility. BW25113, Δ*ytfB*, Δ*ytfB* pGEN and Δ*ytfB* pGEN_ytfB were grown to late exponential phase before inoculation into soft agar to observe motility. Plates were incubated for 12 hours and motility was determined by measuring the diameter of the leading edge of growth. The data are averages of four biological replicates, and error bars represent SEM. Diameters of growth are displayed as a percentage of motility compared to wild type BW25113, whose motility is represented as a dashed line. (**A**) This assay was repeated with UTI89 and UTI89Δ*ytfB*, with the average diameters of growth for six biological replicates displayed as a percentage of motility relative to wild type UTI89. (**B**) P-values were determined using an unpaired student T-test.

Motility and adherence are regulated inversely during infections, so it is thought that bacteria expressing flagella will not express adherence factors, and vice versa^32,33^. Since YtfB is required for binding to kidney cells, we investigated the Δ*ytfB *mutant for motility abnormalities compared to its isogenic wild type. Using a soft agar motility assay, we observed that BW25113Δ*ytfB* displayed a modest but statistically significant increase (11%) in motility compared to the wild-type (Fig. 7). This increased motility in BW25113Δ*ytfB* was restored to wild-type levels upon complementation with *ytfB *on a plasmid (Fig. 7; pGEN-*ytfB*). Increased motility was also observed in UTI89Δ*ytfB*, albeit to a lesser, but still significant, degree (9%; Fig. 7). This is likely due to the higher number of encoded virulence factors, including motility factors, associated with pathotypes which may result in redundancy of function upon deletion of *ytfB*. Nonetheless, our data are consistent with YtfB having a role in regulating the switch between motile and sessile lifestyles in response to changing environments.

## Discussion

YtfB was initially discovered through a genome-wide screen to identify novel cell division genes and regulators in *E. coli*^8^. Misexpression of *ytfB* results in division inhibition, resulting in filamentous cells, which has also been confirmed in this study and others^9^. YtfB has also been shown to localise to midcell in an FtsZ-dependent manner^9^. Midcell localisation is mediated by the LysM-like (OapA) domain which binds preferentially to denuded glycans. Interestingly, the LysM-like domain was not necessary for septal localisation, suggesting that other structural features of YtfB may be important for septal localisation, whilst the LysM-like domain may play additional role(s) in glycan binding, as discussed below.

Here we have shown that the C-terminal domain of YtfB, which includes the LysM-like domain, can bind to N’acetylglucosamine and mannobiose glycan moieties with high affinity. The ability of YtfB to bind to N’acetylglucosamine glycans is not surprising as LysM domains are well characterised to bind to GlcNAcs^10^, and this correlates with the binding to denuded glycans (composed of alternating residues of β-(1,4) linked N-acetylglucosamine [GlcNAcs] and N-acetylmuramic acid [NAM]) previously observed^9^. However, the ability of LysM domains to bind to mannosylated glycans has not previously been reported and raises an interesting possibility that domains may have the ability to bind to other glycans, in addition to their characterised ligands, to adapt to various environmental niches. This is plausible as LysM domains are present in several proteins that have been implicated in virulence such as P60 from *Listeria monocytogenes*^34^ and intimin from enterohaemorragic and enteropathogenic *E. coli*^35^, whilst some LysM-containing proteins are directly involved in adherence to host cells^36–38^. Although there is precedent for LysM-containing proteins to be important for host cell adherence, as we have also demonstrated for YtfB in this study, it also raises the possibility that YtfB may not possess a classical LysM domain. LysM domains are typically ~40 amino acids long, but the LysM-like (OapA) domain of YtfB is much longer at 92 residues. YtfB shows good homology to the LysM consensus sequence^39^, particularly across the N terminus, but does not contain the conserved Ile/Leu and Asn present in the central region of the domain. YtfB was annotated as containing a LysM-like domain based on computational structural similarities to a variety of LysM domains (Pfam01476) but this has not been experimentally confirmed. We also cannot rule out the possibility that other parts of the C-terminal domain of YtfB, other than the LysM-like domain, are involved in glycan binding. A full understanding of the molecular interactions of YtfB with glycans and/or other molecules can only be determined with high resolution structural characterisation of these domains.

Here we present the most comprehensive glycan binding profile of the UPEC strain UTI89, and show that it can bind to a wide range of glycans, from simple to highly complex chemical structures. The ability of UPEC to adhere to glycans is important for its adaptation to the diverse environmental niches it encounters such as the gastrointestinal tract, bladder lumen and kidney where a wide range of glycans are present on host cell surfaces. In this study, we have shown that YtfB has high affinity for specific mannobiose moieties, which have been well characterised for binding of UPEC to host tissue during the establishment of urinary tract infections within the bladder^40^, whilst mannose glycans are the most prominent glycans reported on kidney (HEK-293) cells^41^. Loss of YtfB also resulted in a significant reduction in adhesion of UTI89 to human kidney (HEK-293) cells and a reduced fitness for growth in human urine. Taken together it is possible the YtfB is important for adhesion during ascending UTIs. Many adhesins have been described for UPEC which remain uncharacterised and are redundant in murine urinary tract infection models^42^ in addition to well characterised adhesins specific for bladder cell (type 1 fimbriae^43^); and kidney cell (Pap pili^44^) adhesion. It should be noted that characterisation of HEK-293 cells has detected expression of markers of several tissues, suggesting that this cell line may not be fully representative of kidney epithelia^45^. This may explain why a phenotype for the *ytfB* mutant was not detected in *vivo*, and highlights the importance of understanding the interconnection between YtfB and both characterised and uncharacterised adhesins, in a range of different tissue types.

One of the critical infective stages of bladder cells for UPEC involves morphological plasticity. During intracellular growth there is a mixture of subpopulations containing both small coccoid cells, and long filamentous cells that are inhibited for division^46^. It is intriguing to us that, although in this study we have described an adherence function for YtfB, we originally identified that expression of this gene causes filamentation^8^. DamX is a cell division protein that is critical for the reversible filamenation observed during UTIs^19^, and causes filamentation when expressed^8,47^. YtfB has many similar characteristics to DamX, including the presence of a glycan-binding domain (SPOR for DamX^48^), and a filamentous phenotype when deleted in combination with *dedD*^9^. What is still not clear is the nature of the link between the function of YtfB as a cell division protein and in adhesion, if any. Future studies to understand this connection during infection, particularly during ascending urinary tract infections, as well as the role of cell division proteins such as DamX and DedD in binding to other glycans, will give insights into the prospect of cell division proteins with potential ‘moonlighting’ functions. Indeed, many proteins involved in maintenance of cell growth e.g. metabolism and protein synthesis^49^, have recently been shown to also function as adhesins to host receptors.

In this study we have shown that YtfB binds to specific glycans and has a role in adhesion to kidney cells, which may suggest a role in establishing infection within a host. We initially identified YtfB in this study due to its homology to *H. influenzae *OapA, which is involved in adherence to epithelial cells during a respiratory infection. Additionally, this protein has previously been shown to localise to the division site in *E. coli*. Thus, although homology can give inference about protein function, it is clear that there may be adaptions of function based on the environmental niche of the bacteria, as well as the possibility of a protein having complementary, or additional functions that need to experimentally investigated. Indeed, YtfB is conserved in Enterobacteriaceae, but it is still unclear if the primary function of YtfB in this group of bacteria is in cell division or binding to glycans present on environmental surfaces (either within a host during infection, or in the environment such as water systems), and if these functions are mutually exclusive. Thus, there is still much to learn about protein function in the context of different environmental conditions.

## Experimental Procedures

### Phylogenetic tree construction

Taxonomic homologues were identified using JackHMMER^14^ with YtfB from *E. coli* MG1665 (accession number NP_418627) as the query sequence. Representative protein sequences were identified using PSI-BLAST^13^ from the following taxids: *Enterobacteriaceae*(543); *Yernsiaceae* (1903411); *Erwiniaceae* (1903409); *Pectobacteriaceae* (1903410); *Morganellaceae *(1903414); *Hafniaceae* (1903412); *Moraxellaceae* (468); *Vibrionaceae* (641); *Pasteurellaceae* (712); *Moritellaceae* (267891); *Ferrinmonadaceae* (267892); *Campylobacteriaceae* (72296); *Streptococcaceae* (1300); *Mycobacteriaceae* (1762). Protein sequences were aligned using ClustalW and a phylogenetic tree was generated using MEGA X^15^.

### Growth conditions

Bacteria were cultured in lysosgeny broth (LB; 1% tryptone, 0.5% yeast extract, 1% NaCl), at 37°C either with shaking or statically. Human urine was collected from a male donor and filter sterilised.

### Strains

All strains used are listed in Supplementary Table 3. Chromosomal deletions of *ytfB* were generated using lambda Red recombination^50^. The FLP recombinase (pCP20) was used to mediate removal of the kanamycin resistance maker, leaving a frt scar^51^. Gene deletions were verified by colony PCR. BW25113 with a C-terminal FLAG fusion to *ytfB* under the native promoter was also made by lambda Red recombination, using a primer with homology to the 3’ of the *ytfB* ORF (ALB268) and a reverse primer with homology ~30 bp downstream of the stop codon (ALB265). The FLAG tag sequence (GAC TAC AAG GAC GAT GAC GAC AAA) was included in primer ALB268 for incorporation via PCR amplification. Correct construction of BW25113 ytfB-FLAG was verified by Sanger sequencing at the Australian Genome Research Facility.

### Plasmid construction

All plasmids and primers are listed in Supplementary Tables 3 and 4 respectively. The complementation plasmid pGEN-ytfB was constructed by PCR amplifying the *ytfB* gene and corresponding promoter region (100 nucleotides upstream and ORF) using primers EP1 and EP2. The 739-bp product was cut with EcoRI and BamHI and ligated into the same sites of pGEN-MCS^33^. For purification of the extracellular domain of YtfB, pETMCSIII-*ytfB* was constructed by amplifying the corresponding region (residues 52-212) with primers ALB325 and ALB326. The resulting 486-bp PCR product was cut with NcoI and NdeI and ligated into the same sites of pETMCSIII^52^. Correct inserts were verified by Sanger sequencing at the Australian Genome Research Facility.

### Cell fractionation

Separation of BW25113 *ytfB-FLAG* cellular fractions was performed using an adapted version of Method 4 from Thein *et al*.^53^. Briefly, BW25113 *ytfB-FLAG* was grown in 3 L of LB at 37 °C with shaking until an OD_600_ of 0.8 was reached. An aliquot was collected to represent the whole cell lysate, and cells were harvested by centrifugation. The resulting pellet was resuspended in 30 ml 0.2 mM Tris pH8, 1 M sucrose, 1 mM EDTA, and lysozyme was added to a final concentration of 1 mg/ml. Cells were incubated at room temperature for 5 min to allow lysis to begin. The mixture was then gently swirled and 120 ml dH_2_0 was added before being placed on ice. An aliquot of cells were checked visually using a phase contrast microscope to observe spheroblasting. Cells were centrifuged at 200 000 × g for 45 min at 4 °C, and the resulting supernatant represents the periplasmic fraction. The pellet was resuspended in 22 ml ice-cold 10 mM Tris pH7.5, 5 mM EDTA, 0.2 mM DTT supplemented with 50 µl 1 mg/ml DNase and cells were lysed by sonication. Unbroken cells were removed by centrifugation at 4000 × g for 10 min at 4 °C. The supernatant was centrifuged at 300 000 × g for 3 h at 4 °C to separate fractions into cytoplasm (supernatant) and crude membranes (pellet). The membrane pellet was resuspended in 27 ml 50 mM Tris pH8, 2% Triton X-100, 10 mM MgCl_2_ and centrifuged at 85 000 × g for 30 min at 4 °C. The supernatant contains the inner membrane fraction. The remaining outer membrane pellet was washed once in 22 ml 50 mM Tris pH8, 2% Triton X-100, 10 mM MgCl_2_ and 3 times in 22 ml dH_2_O, with centrifugation steps of 85 000 × g for 20 min at 4 °C, before resuspension in 22 ml dH_2_O. All fractions were stored at −20 °C. Volumes of each fraction, equivalent to an equal proportion of the original culture volume, were separated by SDS-PAGE. Western blotting was performed as previously described, using mouse α-FLAG antibodies at 1:2500. As a control for efficient cell fractionation, the membrane was also probed with rabbit α-FtsZ antibodies at 1:10 000, rabbit α-OmpA antibodies at 1:100 000 and rabbit α-MBP antibodies at 1:3 000.

### Protein purification

His_6_-YtfB was produced and purified from the overproduction *E. coli* strain BL21 AI (Invitrogen). Strains were grown overnight in LB containing ampicillin before being diluted in fresh media to a starting optical density (OD_600_) of 0.05. Cultures were incubated at 37 °C with shaking until the OD_600_ reached 0.4. Arabinose was added to a final concentration of 0.2% to induce protein expression, and cultures were incubated for a further 4 h before cells were harvested by centrifugation. The pellet was resuspended in lysis buffer (25 mM Tris pH 7.6, 2 mM DTT, 1 mg/ml lysozyme, 50 µg/ml DNAse, Roche complete protease tablet), and incubated at 25 °C for 1 h. Cell lysis was performed by freeze/thaw in liquid nitrogen, and the sample was sonicated and centrifuged at high speed for 30 min 4 °C. Supernatant containing soluble protein was filtered using a 0.2 µm syringe followed by protein capture using a Nickel Affinity HisTrap column and AKTA system (GE Healthcare). Anion ion exchange chromatography was performed to increase purity of the protein using a MonoQ column (GE Healthcare). His_6_-YtfB was dialysed into buffer containing 20 mM TrispH 8.0, 0.2 M NaCl, 10% glycerol. Concentration was calculated by measuring UV absorbance at 280 nm. Protein purity was determined to be ~95% based on visual judgement of Coomassie brilliant blue stained protein separated by SDS-PAGE.

### Glycan microarrays

#### Printing of array slides

Arrays were printed as previously described by Day *et al*. ^54^. Briefly, 415 glycans were printed at a concentration of 500 µM using an ArrayJet Argus Marathon non-contact printer, whereby four drops per spot was spotted in quadruplicate onto a CaptialBio OPEpoxy glass slide. After the printed slides were neutralised, slides were blocked for 5 mins in 0.5% BSA in PBS. The slides were then rinsed in PBS and dried by centrifugation at 200 × g for 4 mins.

#### Application of purified protein

1–3 µg of his_6_-YtfB was complexed with a mouse α-his antibody, and Alexa Fluor647 labelled rabbit α-mouse and goat α-rabbit antibodies at a ratio of 4:2:1 in array PBS (PBS with 1.8 mM MgCl_2_/CaCl_2_) to a final volume of 300 µl. The antibody-protein mix was incubated on ice for 10 min prior to addition to the array for 15 min at room temperature in the dark. The slide was then immersed in array PBS and washed briefly for two min with gentle shaking. The slide was then transferred to a clean 50 mL tube, to dry by centrifugation at 200 × g for 4 min.

#### Application of whole cells

*E. coli* UTI89 and Δ*ytfB *mutant were grown at 37 °C overnight and harvested into sterile PBS. The cells were then labelled with Bodipy-methylester Lipophilic counterstain, by using 1 µL of 1 mM Bodipy into a 500 µL cell suspension. The cells were labelled for 30 mins, then washed in PBS twice to remove excess dye. The cells were resuspended in a final volume of 200 µL before being diluted 1:3 in array PBS and placed onto a glycan array slide with a 65 µL geneframe fitted. The cells were added directly to the slide surface without prior blocking. The cells were incubated on the array for 15 mins at room temperature in the dark. After the incubation time, the slide was then immersed in array PBS and washed briefly for two min with gentle shaking. The slide was then transferred to a clean 50 mL tube, to dry by centrifugation at 200 × g for 4 min.

#### Analysis of binding

The glycan slide incubated with either protein or whole cells was scanned using an Innopsys Innoscan 1100AL microarray scanner, using the 488 nm, 532 nm and 635 nm lasers, using low laser power and 50% PMT gain settings. The acquired image was then analysed using the Mapix software, overlaying the image with the map (Gal) file. The experiment was repeated three times, and binding was classified as positive when the average RFU (relative fluorescence units) of a specific structure had a value above mean background (defined as the average background fluorescence plus 3 standard deviations), and had a P value of < 0.005 (student’s T-Test). For the purified protein experiment, a fluorescent antibody complex of mouse anti-His, rabbit anti-mouse IgGAlexa Fluor647 and goat anti-rabbit IgG Alexa Fluor647 was also performed as a control to remove any glycan structures which may have been attributed to the detection antibodies. For this control, the slide was treated the same way as outlined above, and results showed that no glycan structures were bound by the antibody complex.

### Protein-glycan affinity determination by surface plasmon resonance

Steady state affinity of his6-YtfB to a subset of glycans was determined by single cycle kinetics using a GE Healthcare BIAcore S200 or T200 instrument. The his6-YtfB protein was immobilized onto a CM5 series S sensor chip using amine coupling, in which the carboxylmethyl dextran matrix of the sensor chip was activated by a 720 sec injection of a mixture of 0.2 M 1-ethyl-3-[(3-dimethylamino)propyl]-carbodiimide (EDC) and 0.05M N- hydroxysuccinimide (NHS) which resulted in the conversion of carboxyl groups to an NHS ester. The proteins were diluted in 10 mM sodium acetate buffer pH 4.0 at a concentration of 100 µg/mL and flowed over the surface for 600 sec at 10 µL/min. The remaining unreacted NHS ester groups were neutralized by an injection of 1 M ethanolamine-HCl (pH 8.0). Under these conditions 1,800 RU of YtfB was immobilized on flow cell 2, 3 and 4. Flow cell 1 was a negative control, where the flow cell underwent the same treatment as the active flow cells, without the immobilization of the protein. This enables reference cell subtraction of the responses (2–1, 3-1, 4-1). The optimal pH for immobilization of the protein was determined by performing a pH scout with 10 mM sodium acetate solutions from pH 4.0 to pH 5.5. Glycan stocks of 4–10 mM were made up in sterile milliQ water and further diluted in the BIAcore running buffer, PBS. In each cycle, five or six dilutions of glycan were flowed over the immobilized protein at concentrations ranging from 3.2 nM to 10 µM, ata flow rate of 30 µL/min. A series of PBS buffer only controls enables blank subtraction of the reference subtracted sensorgrams.

### Static infection of HEK cells

The human embryonic kidney cell line HEK-293 (ATCC® CRL-1573TM) was cultured to confluence in 24-well cell culture plates at 37 °C in 5% CO_2_ in DMEM 10% FBS. Bacterial strains were grown statically overnight at 37 °C in 5 ml LB to promote fimbrial expression. Bacterial cultures were diluted to 10^9^ cells/ml (OD_600_ = 1). To infect HEK cells, 0.1 µl/mm^2^ of bacterial culture was added to each well, to give a MOI of 100. Cell culture plates were centrifuged for 5 min, 600 g at 24 °C to force the bacteria onto the HEK cell surface then incubated at 37 °C with 5% CO_2_ for 2 hours. Following incubation, cells were washed with PBS 6 times. To lyse the HEK cells, 1 ml of lysis solution (0.5% Trypsin-EDTA, 0.1% Triton X-100) was added and incubated for 15 minutes at 37 °C in 5% CO_2_, and bacterial cells were resuspended by pipetting. Dilutions of the bacteria were plated onto LB agar plates to count the CFU/ml. This assay was repeated eight times with two technical replicates for each assay. Averages of technical replicates were calculated and the data was normalised to the wild type control before averages and standard errors of the means were calculated for the biological replicates. Unpaired student T-tests were then performed to test for significant differences between strains.

### Yeast agglutination assay

Performed as described^55^, with the following additions. Briefly, a single colony of strains UTI89, UTI89Δ*ytfB* and UTI89Δ*fimH* (a gift from JakobMøller-Jensen;negative control) were inoculated in 20 ml LB at 37 °C and grown statically for 24 h. Cells were centrifuged and resuspended in PBS without D-mannose and in PBS with 3% D-mannose to approximately OD_600_ = 2. Cultures were serially diluted 2-fold before 100 µl of each sample was added into 24 well plates. 20 µl of 3% baker’s yeast suspension was then added to each well. Yeast aggregation was observed visually and the agglutination titre of the most diluted bacterial sample giving a positive agglutination reaction at room temperature after 10 min was recorded. Technical and biological replicates were performed twice.

### Motility assay

Motility was observed using soft-agar plates as previously described^56^.

### Ethic statement

All procedures were approved and performed in accordance with the Institutional Animal Care and Use Committee (IACUC) in Nanyang Technological University, School of Biological Sciences and (ARFSBS/NIEA0247) for CAUTI model.

## Supplementary information

Supplementary data and Experimental procedures.

## Data Availability

All data generated or analysed during this study are included in this published article (and its Supplementary Information files).
