# Supplementary material for: The novel E. coli cell division protein, YtfB, plays a role in eukaryotic cell adhesion
Source: Sci Rep. 2020 Apr 21;10:6745. doi: 10.1038/s41598-020-63729-7 (PMC7174318; doi:10.1038/s41598-020-63729-7)
Supplement: Supplementary file 1 — Supplementary data and Experimental procedures. [file 41598_2020_63729_MOESM1_ESM.pdf]

<u>The novel *E. coli* cell division protein, YtfB, plays a role in eukaryotic cell adhesion</u>

Amy L. Bottomley[1*], Elizabeth Peterson[1], Gregory Iosifidis[1], Adeline Mei Hui Yong [2], Lauren E. Hartley-Tassell[3], Shirin Ansari[1], Chris McKenzie[1], Catherine Burke[1, 4], Iain G. Duggin[1], Kimberly A. Kline[2], Elizabeth J. Harry[1].

<u>Supplementary data and Experimental procedures</u>

Supplementary Figure 1

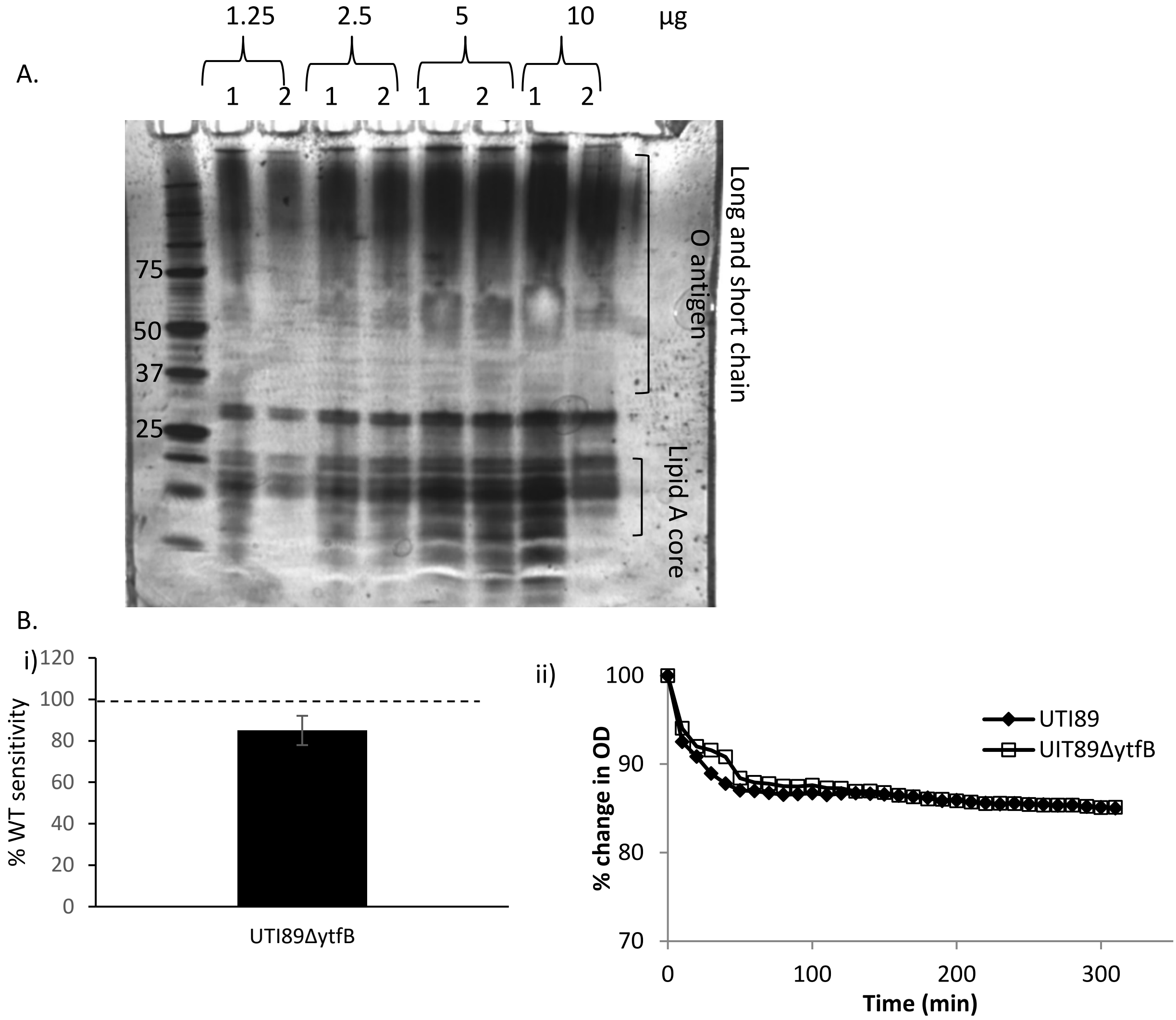

No difference in LPS profile and lysozyme sensitivity of UTI89 and UTI89 Δ*ytfB*

A. Lipopolysaccharide (LPS) was extracted from overnight cultures of UTI89 (1) and UTI89 Δ*ytfB* (2), and increasing concentrations were separated using a 4-12% SDS gel before being silver stained. No difference in profile was observed between wild type and Δ*ytfB* mutant. Protein standards are shown in kDa.
B. Sensitivity of UTI89 and UTI89 Δ*ytfB* to lysozyme was measured using a sold medium assay (i) or in liquid (ii) and showed no difference in sensitivity between wild type and mutant. For the solid assay, 0.5 mg/ml lysozyme was spotted onto agar containing a bacterial suspension and zones of lysis were recorded. Lysozyme sensitivity was normalised to that of UTI89. For the liquid assay, bacterial suspensions were incubated with 10 mg/ml lysozyme with shaking at 37 °C and a reduction in optical density was recorded over time. Experiments were performed with at least two biological replicates and error bars represent the standard error of mean.

Supplementary Figure 2

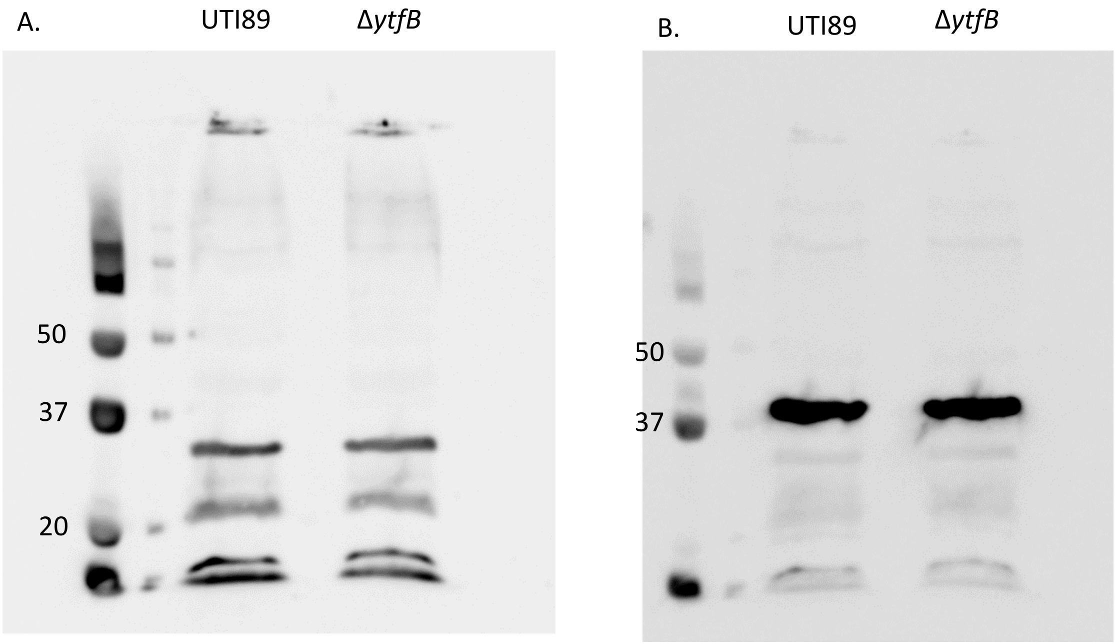

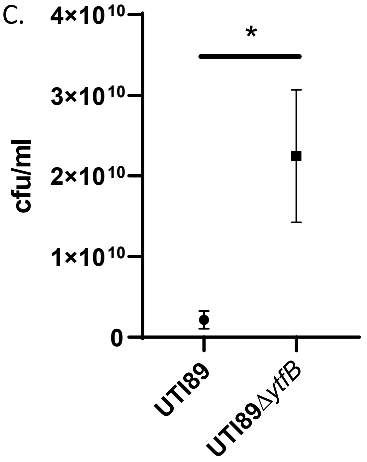

Strain	Average agglutination titre	Average cfu/ml to agglutinate yeast
**UTI89**	1 in 32	2.15×10^9
**UTI89Δ*ytfB***	1 in 16	2.25×10^{10}
**UTI89Δ*fimH***	No agglutination	n/a
**Competitively inhibited by D-mannose**		
**UTI89**	No agglutination	n/a
**UTI89Δ*ytfB***	No agglutination	n/a
**UTI89Δ*fimH***	No agglutination	n/a

Fimbrial profiles of UTI89 and Δ*ytfB*

Wild-type UTI89 and the isogenic mutant Δ*ytfB* were grown in LB medium overnight statically to promote fimbrial expression. Cell concentrations were normalised and whole cell lysates were probed with α-fim antibodies (A) at 1:500 to detect the absence or presence of fimbriae. Lysates were also probed with FtsZ antibodies (B) at 1:10 000 on a separate blot as a loading control. Protein standards are labelled as kDa. Yeast aggregation of 2-fold dilutions of bacterial cultures were observed visually and the agglutination titre of the most diluted bacterial sample giving a positive agglutination reaction at room temperature after 10 min was recorded (C). The cfu/ml of positive wells was calculated and used to calculate the corresponding cfu needed to agglutinate yeast. The data is an average of two biological replicates; error bars represent SEM. Asterisks indicate P-value <0.05 as determined by an unpaired student T-test.

Supplementary Figure 3

A.

i)

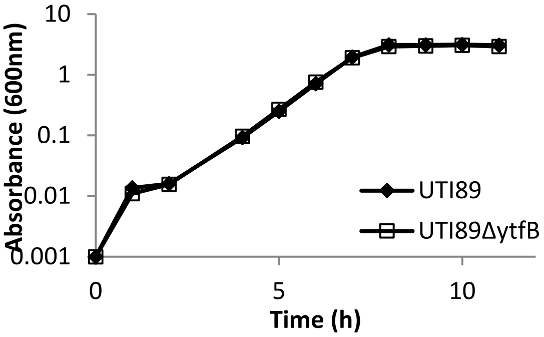

B.

i)

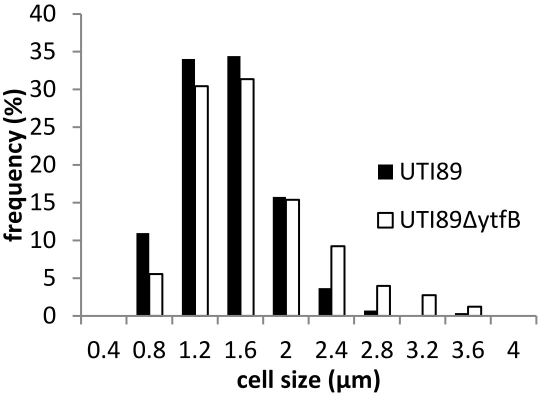

ii)

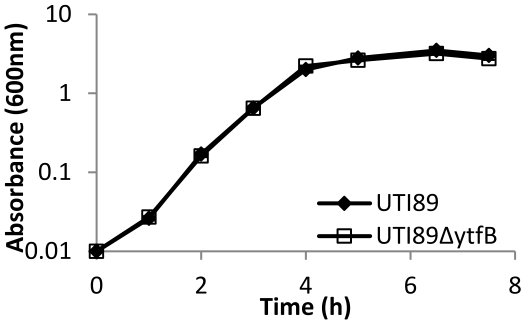

ii)

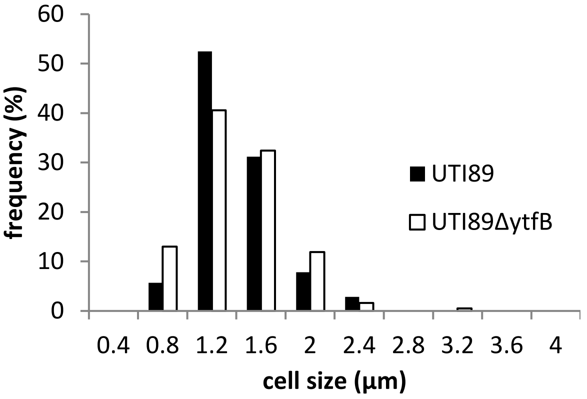

C.

i)

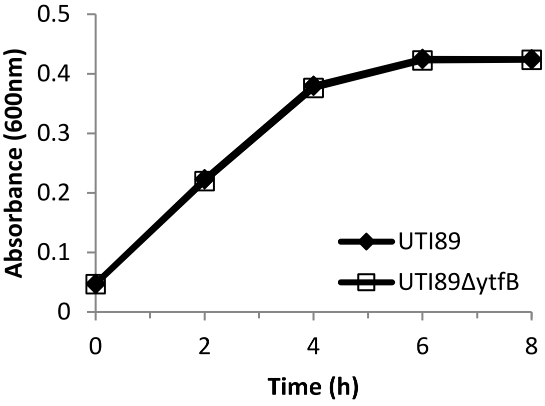

D.

i)

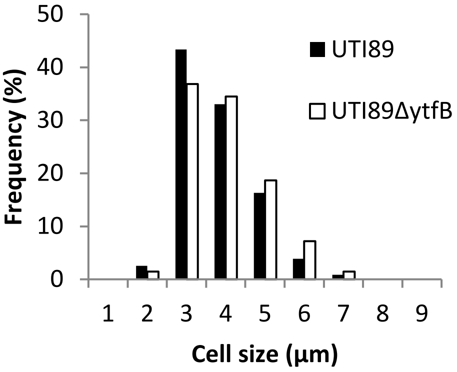

ii)

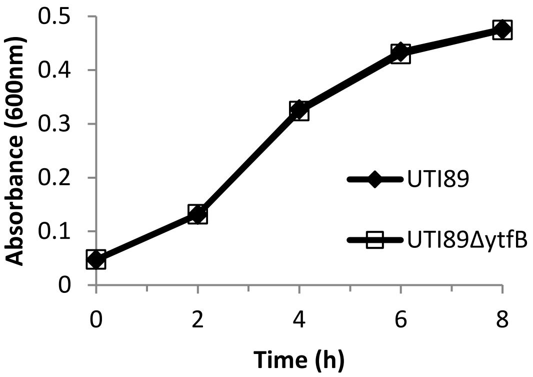

ii)

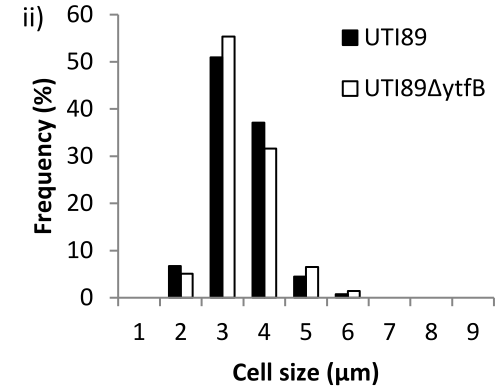

iii)

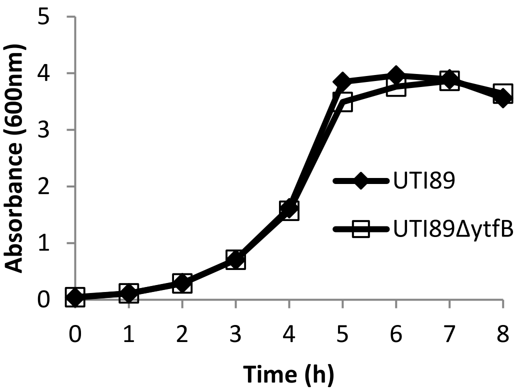

iii)

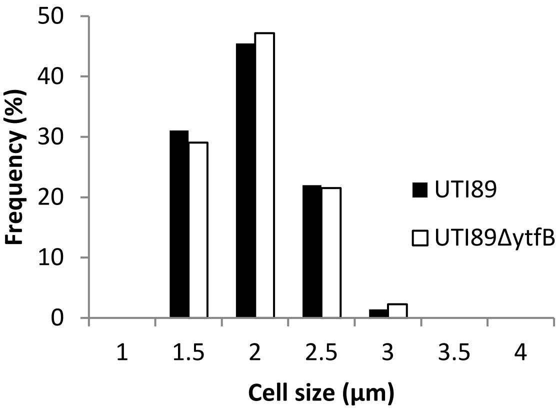

<u>Growth and cell size of UTI89 and UTI89Δ*ytfB* in various media</u>

Growth of UTI89 and the isogenic mutant <u>Δ*ytfB*</u> was observed over time in minimal medium with glucose as the carbon source (A i), synthetic human urine (A ii), dilute human urine (C i), concentrated human urine (C ii) or minimal medium containing 10% fetal bovine serum (Ciii). At mid-exponential growth, samples were taken to observe cell morphology under the microscope. Cell lengths were measured (n>100) for each growth condition: minimal medium with glucose as the carbon source (B i), synthetic human urine (B ii), dilute human urine (D i), concentrated human urine (D ii) or minimal medium containing 10% fetal bovine serum (D iii). No differences were observed in growth rates and cell length under any growth condition.

Supplementary Figure 4

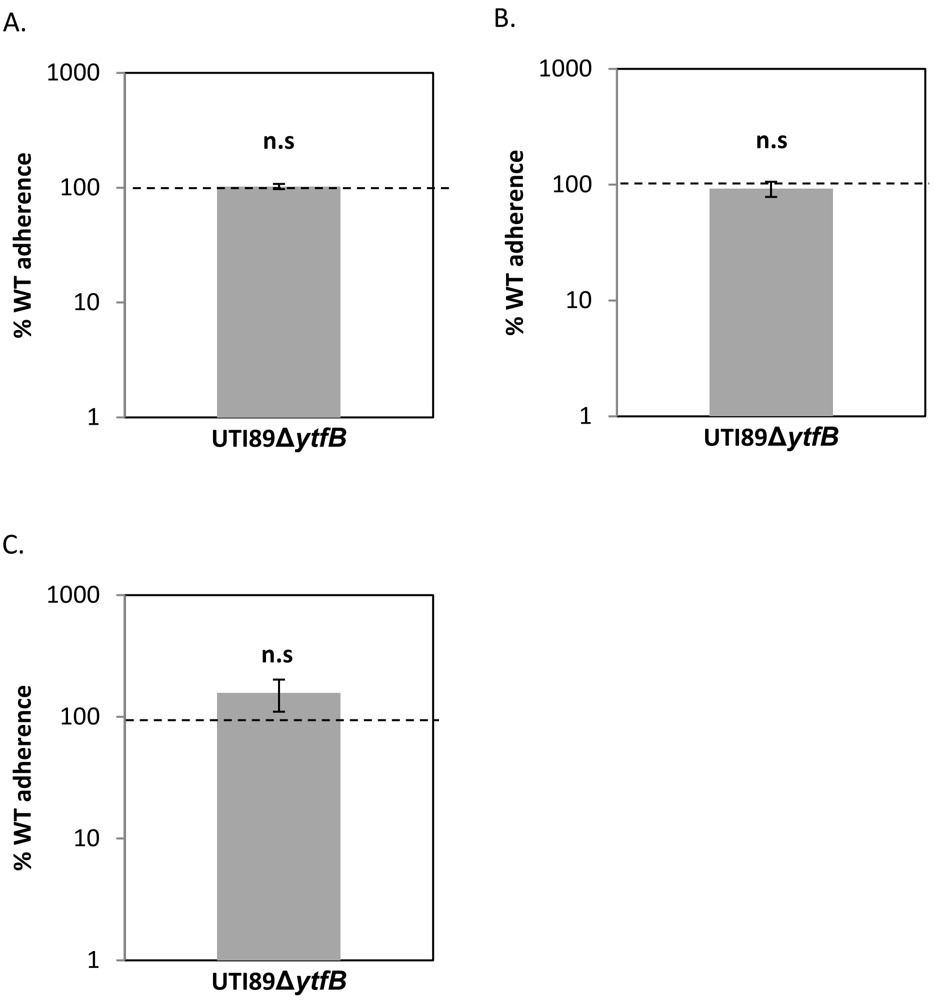

YtfB is not required for *in vitro* bladder cell adhesion, invasion or intracellular growth.

Statically grown UTI89 and UTI89Δ*ytfB* incubated with a monolayer of PD07i bladder epithelial cells and adherence (A), invasion (1h intracellular growth) (B) or intracellular growth (24h intracellular growth) (C) measured. The data are averages of at least three biological replicates, and error bars represent SEM. P values were determined using an unpaired student T test. Adherence is displayed as a percentage compared to wild type UTI89, whose adherence, invasion or growth is represented as a dashed line.

Supplementary Figure 5

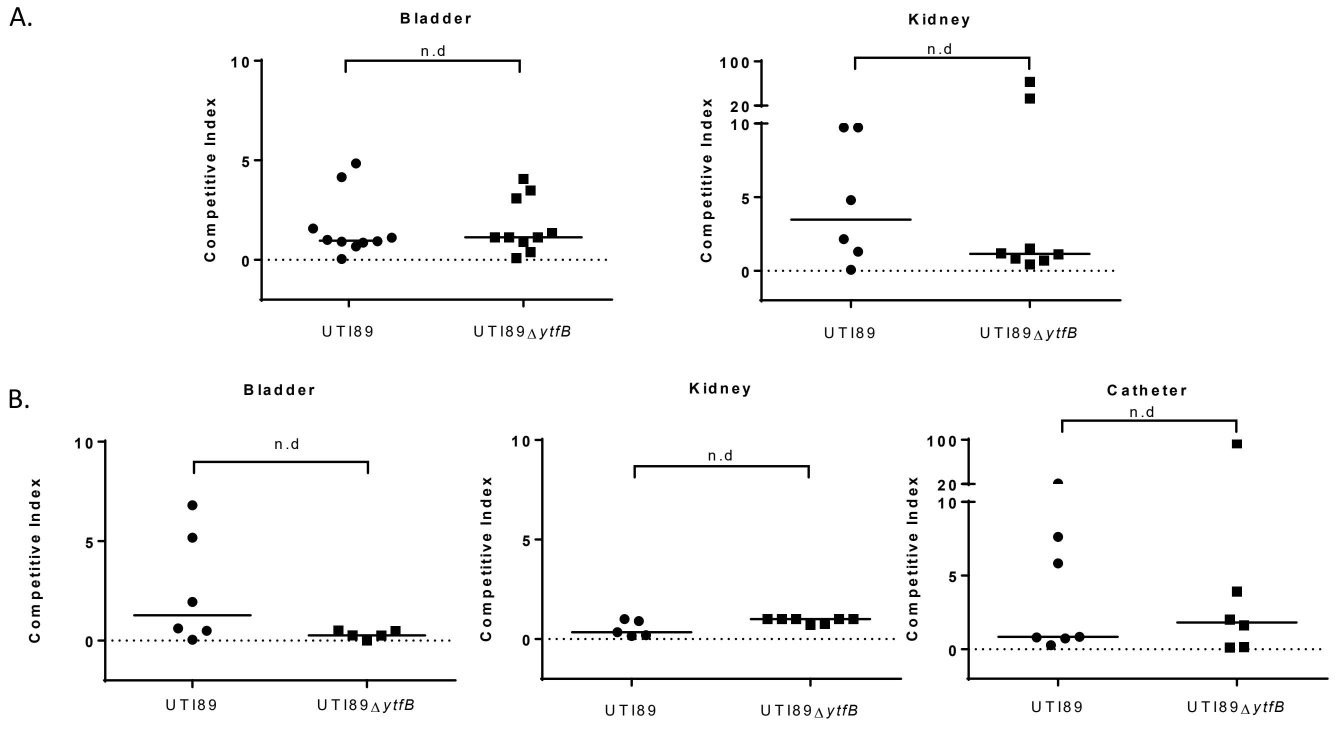

YtfB is not required *in vivo* using competitive UTI and CAUTI infections.

The mice were infected with either an ascending UTI (A) or catheter-associated UTI (B) with a 1:1 ratio of *E. coli* strains UTI89: UTI89*ytfB::kan* (labelled as UTI89 in the figure) or UTI89*ΔytfB:* UTI89*ytfB::kan* (labelled as UTI89*ΔytfB* in the figure) at 10^7 colony forming units per inoculum, harvested at 24 hours post infection, and the recovered bacteria were enumerated on selective media for each strain. The competitive index (CI) was calculated as described in the materials and methods. Each dot represents one mouse, and the solid horizontal lines indicate the median. The limit of detection (LOD) of 40 CFU is indicated by the dotted line. Two biological replicates were carried out for each infection model, with 3-5 mice per experiment for both competitive infections. Mice that lost the catheter or died prior to the time of sacrifice were omitted. Statistical analysis was performed using the Mann-Whitney test. (n.d) No difference.

#	Structure	WT UT189	UTI89 Δ*YtfB*	rYtfB-His	#	Structure	WT UT189	UTI89 Δ*YtfB*	rYtfB-His	#	Structure	WT UTI89	UTI89 Δ*YtfB*	rYtfB-His	#	Structure	WT UTI89	UTI89 Δ*YtfB*	rYtfB-His
	**Monosaccharides**				**Terminal Galactose**					**Fucosylated**					**Sialylated**				
1	Fucα-sp3				75	Galα1-2Galβ-sp3				71	Fucα1-2Galβ-sp3				169	Neu5Acα2-3Galβ-sp3			
2	Galα-sp3				76	Galα1-3Galβ-sp3				72	Fucα1-3GlcNAcβ-sp3				170	Neu5Acα2-6Galβ-sp3			
3	Galβ-sp3				77	Galα1-3GalNAcβ-sp3				73	Fucα1-4GlcNAcβ-sp3				171	Neu5Acα2-3GalNAcα-sp3			
4	GalNAcα-sp0				78	Galα1-3GalNAcα-sp3				215	Fucα1-2Galβ1-3GlcNAcβ-sp3				172	Neu5Acα2-6GalNAcα-sp3			
5	GalNAcα-sp3				80	Galα1-3GlcNAcβ-sp3				216	Fucα1-2Galβ1-4GlcNAcβ-sp3				174	Neu5Gcα2-6GalNAcα-sp3			
6	GalNAcβ-sp3				81	Galα1-4GlcNAcβ-sp3				217	Fucα1-2Galβ1-3GalNAcα-sp3				186	Neu5Acα2-8Neu5Acα2-sp3			
7	Glcα-sp3				83	Galα1-6Glcβ-sp4				219	Fucα1-2Galβ1-4Glcβ-sp4				205	Neu5Acα2-6GalNAcβ-sp3			
9	Glcβ-sp3				84	Galβ1-2Galβ-sp3				226	Fucα1-2(Galα1-3)Galβ-sp3				206	Neu5Gcα2-3Gal-sp3			
10	GlcNAcβ-sp3				85	Galβ1-3GlcNaAcβ-sp3				233	Galβ1-3(Fucα1-4)GlcNAcβ-sp3				289	Galα1-3(Neu5Acα2-6)GalNAcα-sp3			
14	GlcN(Gc)β-sp4				87	Galβ1-3Galβ-sp3				234	Fucα1-3(Galβ1-4)GlcNAcβ-sp3				290	Galβ1-3(Neu5Acα2-6) GalNAcα-sp3			
15	$HOCH_2(HOCH)_4CH_2NH_2$				88	Galβ1-3GalNAcβ-sp3				235	Fucα1-2(GalNAcα1-3)Galβ-sp3				292	Neu5Acα2-3Galβ1-3GalNAcα-sp3			

16	Manα-sp3				89	Galβ1-3GalNAcα-sp3				287	3-O-Su-Galβ1-3(Fucα1-4)GlcNAcβ-sp3				293	Neu5Acα2-3Galβ1-4Glcβ-sp3			
18	Manβ-sp4				93	Galβ1-4Glcβ-sp4				288	Fucα1-3(3-O-Su-Galβ1-4)GlcNAcβ-sp3				294	Neu5Acα2-3Galβ1-4Glcβ-sp4			
19	ManNAcβ-sp4				94	Galβ1-4Galβ-sp4				359	Fucα1-2(Galα1-3)Galβ1-3GlcNAcβ-sp3				295	Neu5Acα2-6Galβ1-4Glcβ-sp2			
20	Rhaα-sp3				97	Galβ1-4GlcNAcβ-sp3				360	Fucα1-2(Galα1-3)Galβ1-4GlcNAcβ-sp3				298	Neu5Acα2-3Galβ1-4GlcNAcβ-sp3			
22	GlcNAcβ-sp4				100	Galβ1-6Galβ-sp4				362	Fucα1-2(Galα1-3)Galβ1-3GalNAcα-sp3				299	Neu5Acα2-3Galβ1-3GlcNAcβ-sp3			
37	3-O-Su-Galβ-sp3				145	Galβ1-3(6-O-Su)GlcNAcβ-sp3				363	Fucα1-2(Galα1-3)Galβ1-3GalNAcβ-sp3				300	Neu5Acα2-6Galβ1-4GlcNAcβ-sp3			
38	3-O-Su-GalNAcα-sp3				146	Galβ1-4(6-O-Su)Glcβ-sp2				364	Fucα1-3(Galα1-3Galβ1-4)GlcNAcβ-sp3				303	Neu5Gcα2-3Galβ1-4GlcNAcβ-sp3			
43	6-O-Su-GlcNAcβ-sp3				147	Galβ1-4(6-O-Su)GlcNAcβ-sp3				366	Fucα1-2(GalNAcα1-3)Galβ1-3GlcNAcβ-sp3				304	Neu5Gcα2-6Galβ1-4GlcNAcβ-sp3			
44	GlcAα-sp3				150	3-O-Su-Galβ1-3GalNAcα-sp3				368	Fucα1-2(GalNAcα1-3)Galβ1-4GlcNAcβ-sp3				306	9-NAc-Neu5Acα2-6Galβ1-4GlcNAcβ-sp3			
45	GlcAβ-sp3				151	6-O-Su-Galβ1-				371	Fucα1-2Galβ1-				315	Neu5Acα2-3Galβ1-4-(6-O-			

						3GalNAcα-sp3					3(Fucα1-4)GlcNAcβ-sp3					Su)GlcNAcβ-sp3			
46	6-H$_2$PO$_3$Glcβ-sp4				152	3-O-Su-Galβ1-4Glcβ-sp2				372	Fucα1-3(Fucα1-2Galβ1-4)GlcNAcβ-sp3				317	Neu5Acα2-3Galβ1-3-(6-O-Su)GalNAcβ-sp3			
47	6-H$_2$PO$_3$Manα-sp3				153	6-O-Su-Galβ1-4Glcβ-sp2				392	Fucα1-2(GalNAcα1-3)Galβ1-3GalNAcα-sp3				318	Neu5Acα2-6Galβ1-4-(6-O-Su)GlcNAcβ-sp3			
48	Neu5Acα-sp3				155	3-O-Su-Galβ1-3GlcNAcβ-sp3				479	Fucα1-2Galβ1-3GlcNAcβ1-3Galβ1-4Glcβ-sp4				319	Neu5Acα2-3-(6-O-Su)Galβ1-4GlcNAcβ-sp3			
49	Neu5Acα-sp9				157	3-O-Su-Galβ1-4GlcNAcβ-sp3				480	Fucα1-2Galβ1-3GlcNAcβ1-3Galβ1-4GlcNAcβ-sp2				321	(Neu5Acα2-8)$_3$-sp3			
52	Neu5Gcα-sp3				159	4-O-Su-Galβ1-4GlcNAcβ-sp3				483	Fucα1-3(Fucα1-2(Galα1-3)Galβ1-4)GlcNAcβ-sp3				323	Neu5Acα2-6Galβ1-3GlcNAc-sp3			
54	9-NAc-Neu5Acα-sp3				161	6-O-Su-Galβ1-3GlcNAcβ-sp3				496	Fucα1-2Galβ1-3(Fucα1-4)GlcNAcβ1-3Galβ1-4Glcβ-sp4				324	Neu5Acα2-6Galβ1-3(6-O-Su)GlcNAc-sp3			
55	3-O-Su-GlcNAcβ-sp3				163	6-O-Su-Galβ1-4GlcNAcβ-sp3				497	Fucα1-3(Fucα1-2Galβ1-4)GlcNAcβ1-3Galβ1-4Glcβ-sp4				331	Neu5Gcα2-3Galβ1-3GlcNAcβ-sp3			
	**Terminal N-Acetylgalactosamine**				176	3-O-Su-Galβ1-4(6-O-Su)Glcβ-sp2				538	Le^{x}1-6'(Le^{c}1-3')Lac-sp4				421	Neu5Acα2-3(GalNAcβ1-4)Galβ1-4Glcβ-sp2			

101	GalNAcα1-3GalNAcβ-sp3				177	3-O-Su-Galβ1-4(6-O-Su)GlcNAcβ-sp2				539	Galβ1-4GlcNAcβ1-6(Fucα1-2Galβ1-3GlcNAcβ1-3)Galβ1-4Glc-sp4				422	Neu5Acα2-3Galβ1-4GlcNAcβ1-3Galβ-sp3			
102	GalNAcα1-3Galβ-sp3				178	6-O-Su-Galβ1-4(6-O-Su)Glcβ-sp2				541	Le^{x}1-6'(Le^{d}1-3')Lac-sp4				423	Fucα1-3(Neu5Acα2-3Galβ1-4)GlcNAcβ-sp3			
103	GalNAcα1-3GalNAcα-sp3				179	6-O-Su-Galβ1-3(6-O-Su)GlcNAcβ-sp2				542	$Le^{c}Le^{x}$1-6'(Le^{c}1-3')Lac-sp4				426	Neu5Acα2-3Galβ1-3(Fucα1-4)GlcNAcβ-sp3			
104	GalNAcβ1-3Galβ-sp3				180	6-O-Su-Galβ1-4(6-O-Su)GlcNAcβ-sp2				543	Le^{x}1-6'(Le^{b}1-3')Lac-sp4				428	Fucα1-3(Neu5Acα2-3Galβ1-4)6-O-Su-GlcNAcβ-sp3			
106	GalNAcβ1-4GlcNAcβ-sp3				181	3,4-O-Su_2-Galβ1-4GlcNAcβ-sp3				7A	Fucα1-2Galβ1-3GlcNAcβ1-3Galβ1-4Glc				429	Fucα1-3(Neu5Acα2-3(6-O-Su)Galβ1-4)GlcNAcβ-sp3			
192	GalNAcβ1-4(6-O-Su)GlcNAcβ-sp3				182	3,6-O-Su_2-Galβ1-4GlcNAcβ-sp2				7B	Galβ1-3(Fuca1-4)GlcNAcβ1-3Galβ1-4Glc				433	Neu5Acα2-3Galβ1-3(Neu5Acα2-6)GalNAcα-sp3			
193	3-O-Su-GalNAcβ1-4GlcNAcβ-sp3				183	4,6-O-Su_2-Galβ1-4GlcNAcβ-sp2				7C	Galβ1-4(Fuca1-3)GlcNAcβ1-3Galβ1-4Glc				434	Neu5Acα2-8Neu5Acα2-3Galβ1-4Glcβ-sp4			
194	6-O-Su-GalNAcβ1-4GlcNAcβ-sp3				184	4,6-O-Su_2-Galβ1-4GlcNAcβ-sp3				7D	Fucα1-2Galβ1-3(Fucα1-4)GlcNAcβ1-3Galβ1-4Glc				527	Neu5Acα2-3Galβ1-4GlcNAcβ1-3Galβ1-4GlcNAcβ-sp2			
195	6-O-Su-GalNAcβ1-4-(3-O-Su)GlcNAcβ-sp3				189	3,6-O-Su_2-Galβ1-4(6-O-Su)GlcNAcβ-sp2				7E	Galβ1-3(Fucα1-4)GlcNAcβ1-3Galβ1-4(Fucα1-3)Glc				528	Fucα1-3(Neu5Acα2-3Galβ1-4)GlcNAcβ1-3Galβ-sp3			

196	3-O-Su-GalNAcβ1-4(3-O-Su)-GlcNAcβ-sp3				201	3,4-O-Su$_2$-Galβ1-4GlcNAcβ-sp3				7F	Fucα1-2Gal				529	Neu5Acα2-6(Galβ1-3)GlcNAcβ1-3Galβ1-4Glcβ-sp4			
197	3,6-O-Su$_2$-GalNAcβ1-4GlcNAcβ-sp3				203	Galβ1-4(6-O-Su)GlcNAcβ-sp2				7G	Fucα1-2Galβ1-4Glc				531	GalNAcβ1-4(Neu5Acα2-8Neu5Acα2-3)Galβ1-4Glc-sp2			
198	4,6-O-Su$_2$-GalNAcβ1-4GlcNAcβ-sp3				220	Galα1-3Galβ1-4Glcβ-sp2				7H	Galβ1-4(Fucα1-3)Glc				532	Neu5Acα2-8Neu5Acα2-8Neu5Acα2-3Galβ1-4Glc-sp2			
199	4,6-O-Su$_2$-GalNAcβ1-4-(3-O-Ac)GlcNAcβ-sp3				222	Galα1-3Galβ1-4GlcNAcβ-sp3				7I	Galβ1-4(Fucα1-3)GlcNAc				533	(Neu5Acα2-8)2Neu5Acα2-3(GalNAcβ1-4)Galβ1-4Glc-sp2			
200	4-O-Su-GalNAcβ1-4GlcNAcβ-sp3				224	Galα1-4Galβ1-4Glcβ-sp3				7J	Galβ1-3(Fucα1-4)GlcNAc				534	Neu5Acα2-3Galβ1-4GlcNAcβ1-3Galβ1-4GlcNAcβ-sp3			
202	6-O-Su-GalNAcβ1-4(6-O-Su)GlcNAcβ-sp3				225	Galα1-4Galβ1-4GlcNAc-sp2				7K	GalNAcα1-3(Fucα1-2)Gal				536	Neu5Acα2-3Galβ1-3GlcNAcβ1-3Galβ1-4Glcβ-sp4			
204	4-O-Su-GalNAcβ1-4GlcNAcβ-sp2				228	Galβ1-2Galα1-4GlcNAcβ-sp4				7L	Fucα1-2Galβ1-4(Fucα1-3)Glc				537	Neu5Acα2-3Galβ1-4GlcNAcβ1-3Galβ1-4Glcβ-sp4			
238	GalNAcβ1-4Galβ1-4Glcβ-sp3				229	Galβ1-3Galβ1-4GlcNAcβ-sp4				7M	Galβ1-3(Fucα1-2)Gal				540	Le^{x}1-6'(6'SLN1-3')Lac-sp4			
389	GalNAcβ1-3Galα1-4Galβ1-4Glcβ-sp3				231	Galβ1-4GlcNAcβ1-3GalNAcα-sp3				7N	Fucα1-2Galβ1-4(Fucα1-3)GlcNAc				10A	Neu5Acα2-3Galβ1-3(Fucα1-4)Glc*N*Ac			
1L	GalNAcα1-O-Ser				232	Galβ1-4GlcNAcβ1-				7O	Fucα1-2Galβ1-3GlcNAc				10B	Neu5Acα2-3Galβ1-			

						6GalNAcα-sp3										4(Fucα1-3)Glc*N*Ac			
2C	GalNAcβ1-3Gal				254	Galβ1-3(GlcNAcβ1-6)GalNAcα-sp3				7P	Fucα1-2Galβ1-3(Fucα1-4)GlcNAc				10C	Neu5Acα2-3Galβ1-3Glc*N*Acβ1-3Galβ1-4Glc			
2D	GalNAcβ1-4Gal				262	Galβ1-3GalNAcβ1-3Gal-sp4				8A	SO_3-3Galβ1-3(Fucα1-4)GlcNAc				10D	Galβ1-4(Fucα1-3)Glc*N*Acβ1-6(Neu5Acα2-6Galβ1-4Glc*N*Acβ1-3)Galβ1-4Glc			
2F	GalNAcα1-3Galβ1-4Glc				264	Galβ1-4Galβ1-4GlcNAc-sp3				8B	SO_3-3Galβ1-4(Fucα1-3)GlcNAc				10E	Neu5Acα2-3Galβ1-3(Neu5Acα2-6)GalNAc			
	**Glucose**				373	Galα1-3Galβ1-4GlcNAcβ1-3Galβ-sp3				8C	Galβ1-3GlcNAcβ1-3Galβ1-4(Fucα1-3)GlcNAcβ1-3Galβ1-4Glc				10H	Neu5Acα2-6Galβ1-3GlcNAcβ1-3Galβ1-4(Fucα1-3)Glc			
110	Glcα1-4Glcβ-sp3				375	Galα1-4GlcNAcβ1-3Galβ1-4GlcNAcβ-sp3				8D	Galβ1-4(Fucα1-3)GlcNAcβ1-6(Galβ1-3GlcNAcβ1-3)Galβ1-4Glc				10I	Galβ1-3GlcNAcβ1-3(Neu5Acα2-6Galβ1-4GlcNAcβ1-6)Galβ1-4Glc			
111	Glcβ1-4Glcβ-sp4				376	Galβ1-3GlcNAcβ1-3Galβ1-4Glcβ-sp4				8E	Galβ1-4(Fucα1-3)GlcNAcβ1-6(Fucα1-2Galβ1-3GlcNAcβ1-3)Galβ1-4Glc				10J	Neu5Acα2-6Galβ1-3GlcNAcβ1-3(Galβ1-4GlcNAcβ1-6)Galβ1-4Glc			
112	Glcβ1-6Glcβ-sp4				377	Galβ1-3GlcNAcβ1-3Galβ1-3GlcNAcβ-sp2				8F	Galβ1-4(Fucα1-3)GlcNAcβ1-6(Fucα1-2Galβ1-3(Fucα1-4)GlcNAcβ				10K	Neu5Acα2-3Galβ1-4Glc*N*Ac			

											1-3)Galβ1-4Glc								
164	GlcAβ1-3GlcNAcβ-sp3				378	Galβ1-3GlcNAcα1-3Galβ1-4GlcNAcβ-sp3				8G	Galβ1-4GlcNAcβ1-3Galβ1-4(Fucα1-3)Glc				10L	Neu5Acα2-6Galβ1-4Glc*N*Ac			
165	GlcAβ1-3Galβ-sp3				379	Galβ1-3GlcNAcβ1-3Galβ1-4GlcNAcβ-sp3				8H	Fucα1-2Galβ1-4(Fucα1-3)GlcNAcβ1-3Galβ1-4Glc				10M	Neu5Acα2-3Galβ1-3GlcNAcβ1-3Galβ1-4Glc			
166	GlcAβ1-6Galβ-sp3				380	Galβ1-3GlcNAcα1-6Galβ1-4GlcNAcβ-sp2				8I	Fucα1-3Galβ1-4GlcNAcβ1-3Galβ1-4(Fucα1-3)Glc				10N	Galβ1-3(Neu5Acα2-6)Glc*N*Acβ1-3Galβ1-4Glc			
240	(Glcα1-4)$_3$β-sp4				381	Galβ1-3GlcNAcβ1-6Galβ1-4GlcNAcβ-sp2				8J	Fuca1-2Galb1-4(Fuca1-3)GlcNAcb1-3(Fuca1-2)Galb1-4Glc				10O	Neu5Acα2-6Galβ1-4Glc*N*Acβ1-3Galβ1-4Glc			
241	(Glcα1-6)$_3$β-sp4				382	Galβ1-3GalNAcβ1-4Galβ1-4Glcβ-sp3				8K	Galβ1-4(Fucα1-3)GlcNAcβ1-6(Galβ1-4GlcNAcβ1-3)Galβ1-4Glc				10P	Neu5Acα2-3Galβ1-3(Neu5Acα2-6)Glc*N*Acβ1-3Galβ1-4Glc			
390	(Glcα1-4)$_4$β-sp4				383	Galβ1-4GlcNAcβ1-3Galβ1-4Glcβ-sp2				8L	Galb1-4(Fuca1-3)GlcNAcb1-6(Galb1-4(Fuca1-3)GlcNAcb1-3)Galb1-4Glc				11A	Neu5Acα2-3Galβ1-4Glc			
391	(Glcα1-6)$_4$β-sp4				385	Galβ1-4GlcNAcβ1-3Galβ1-4GlcNAcβ-sp3				8M	Fuca1-2Galb1-4(Fuca1-3)GlcNAcb1-6(Galb1-4GlcNAcb				11B	Neu5Acα2-6Galβ1-4Glc			

											1-3)Galb1-4Glc								
492	(Glcα1-6)$_5$β-sp4				387	Galβ1-4GlcNAcβ1-6Galβ1-4GlcNAcβ-sp2				8N	Galb1-3GlcNAcb1-3Galb1-4(Fuca1-3)GlcNAcb1-6(Galb1-3GlcNAcb1-3)Galb1-4Glc				11C	(Neu5Acα2-8Neu5Ac)n (n<50)			
502	(Glcα1-6)$_6$β-sp4				388	Galβ1-4GlcNAcβ1-6(Galβ1-3)GalNAcα-sp3				8O	Fucα1-2Galβ1-3GlcNAcβ1-3Galβb1-4(Fucα1-3)GlcNAcβ1-6(Galβ1-3GlcNAcβ1-3)Galβ1-4Glc				18A	Neu5Acα2-3Galβ1-4GlcNAcβ1-3Galβ1-4Glc			
18I	GlcA				401	Galβ1-3GlcNAcβ1-3Galβ1-3GlcNAcβ-sp3				8P	GalNAcb1-3(Fuca1-2)Galb1-4Glc				18K	9-NAc-Neu5Ac			
18J	6-O-(H_2PO_4)-Glc				419	3-O-SuGalβ1-4GlcNAcβ1-3Galβ1-4GlcNAcβ-sp3				9A	Galb1-3(Fuca1-2)Galb1-4(Fuca1-3)Glc				18O	Neu5Gc			
19O	Glcα1-4Glcα1-4Glc				420	4-O-SuGalβ1-4GlcNAcβ1-3Galβ1-4GlcNAcβ-sp3				9B	Galβ1-4GlcNAcβ1-6(Fucα1-2Galβ1-3GlcNAcβ1-3)Galβ1-4Glc				19K	Neu5Acα2-3Galβ1-4(Fucα1-3)GlcNAcβ1-3Gal			
19P	Glcα1-4Glcα1-4Glcα1-4Glc				488	Galβ1-4GlcNAcβ1-3(Galβ1-4GlcNAcβ1-6)GalNAcα-sp3				18D	Galα1-3(Fucα1-2)Galβ1-4Glc				**Terminal N-Acetylglucosamine**				

	**Low molecular weight Carageenan and Glycoaminoglycans (GAGS)**				498	(Galβ1-4GlcNAcβ1-3)3-sp3				18E	GalNAcα1-3(Fucα1-2)Galβ1-4(Fucα1-3)Glc				113	GlcNAcβ1-3GalNAcα-sp3			
12A	Neocarratetraose-41, 3-di-O-sulphate (Na+)				499	Galβ1-4GlcNAcβ1-3(Galβ1-4GlcNAcβ1-6)Galβ1-4GlcNAc-sp2				19J	Galβ1-4(Fucα1-3)GlcNAcβ1-3Gal				114	GlcNAcβ1-3Manβ-sp4			
12B	Neocarratetraose-41-O-sulphate (Na+)				501	Galβ1-3GalNAcβ1-3Galα1-4Galβ1-4Glcβ-sp4				19L	Fucα1-2Galβ1-4(Fucα1-3)GlcNAcβ1-3Gal				115	GlcNAcβ1-4GlcNAcβ-Asn			
12C	Neocarrahexaose-24,41, 3, 5-tetra-O-sulphate (Na+)				504	(A-GN-M)$_2$-3,6-M-GN-GNβ-sp4				19M	Galβ1-3(Fucα1-4)GlcNAcβ1-3Gal				117	GlcNAcβ1-4GlcNAcβ-sp4			
12D	Neocarrahexaose-41, 3, 5-tri-O-sulphate (Na+)				1A	Galβ1-3GlcNAc				19N	Fucα1-2Galβ1-3(Fucα1-4)GlcNAcβ1-3Gal				118	GlcNAcβ1-6GalNAcα-sp3			
12E	Neocarraoctaose-41, 3, 5, 7-tetra-O-sulphate (Na+)				1B	Galβ1-4GlcNAc					**Mannose**				149	GlcNAcβ1-4(6-O-Su)GlcNAcβ-sp2			
12F	Neocarradecaose-41, 3, 5, 7, 9-penta-O-sulphate (Na+)				1C	Galβ1-4Gal				119	Manα1-2Manβ-sp4				167	GlcNAcβ1-4-[HOOC(CH$_3$)CH]-3-O-GlcNAcβ-sp4			
12G	ΔUA-2S-GlcNS-6S				1D	Galβ1-6GlcNAc				120	Manα1-3Manβ-sp4				168	GlcNAcβ1--[HOOC(CH$_3$)CH]-3-O-GlcNAcβ-L-alanyl-D-i-glutaminyl-L-lysine			
12H	ΔUA-GlucNS-6S				1E	Galβ1-3GalNAc				121	Manα1-4Manβ-sp4				246	GlcNAcβ1-2Galβ1-3GalNAcα-sp3			
12I	ΔUA-2S-GlucNS				1F	Galβ1-3GalNAcβ				122	Manα1-6Manβ-sp4				247	GlcNAcβ1-3Galβ1-3GalNAcα-sp3			

						1-4Galβ1-4Glc													
12J	ΔUA-2S-GlcNAc-6S				1G	Galβ1-3Glc*N*Acβ1-3Galβ1-4Glc				123	Manβ1-4GlcNAcβ-sp4				248	GlcNAcβ1-3Galβ1-4Glcβ-sp2			
12 K	ΔUA-GlcNAc-6S				1H	Galβ1-4Glc*N*Acβ1-3Galβ1-4Glc				124	Manα1-2Manα-sp4				250	GlcNAcβ1-3Galβ1-4GlcNAcβ-sp3			
12 L	ΔUA-2S-GlcNAc				1I	Galβ1-4GlcNAcβ1-6(Galβ1-4GlcNAcβ1-3)Galβ1-4Glc				258	Manα1-3(Manα1-6)Manβ-sp4				251	GlcNAcβ1-4Galβ1-4GlcNAcβ-sp2			
12 M	ΔUA-GlcNAc				1J	Galβ1-4GlcNAcβ1-6(Galβ1-3GlcNAcβ1-3)Galβ1-4Glc				495	Manα1-3(Manα1-3(Manα1-6)Manα1-6)Manβ-sp4				252	GlcNAcβ1-4GlcNAcβ1-4GlcNAcβ-sp4			
12 N	ΔUA-GalNAc-4S (Delta Di-4S)				1K	Galα1-4Galβ1-4Glc				5A	GlcNAcβ1-2Man				253	GlcNAcβ1-6Galβ1-4GlcNAcβ-sp2			
12 O	ΔUA-GalNAc-6S (Delta Di-6S)				1L	GalNAcα1-O-Ser				5B	GlcNAcβ1-2Manα1-6(GlcNAcβ1-2Manα1-3)Man				255	GlcNAcβ1-3(GlcNAcβ1-6)GalNAcα-sp3			
12P	ΔUA-GalNAc-4S,6S (Delta Di-disE)				1M	Galb1-3GalNAcα1-O-Ser				5C	Manα1-2Man				395	GlcNAcβ1-3(GlcNAcβ1-6)Galβ1-4GlcNAcβ-sp3			
13 A	ΔUA-2S-GalNAc-4S (Delta Di-disB)				1N	Galα1-3Gal				5D	Manα1-3Man				493	(GlcNAcβ1-4)$_5$β-sp4			
13 B	ΔUA-2S-GalNAc-6S (Delta Di-disD)				1O	Galα1-3Galβ1-4GlcNAc				5E	Manα1-4Man				503	(GlcNAcβ1-4)$_6$β-sp4			
13 C	ΔUA-2S-GalNAc-4S-6S (Delta Di-tisS)				1P	Galα1-3Galβ1-4Glc				5F	Manα1-6Man				505	(GN-M)$_2$-3,6-M-GN-GNβ-sp4			
13 D	ΔUA-2S-GalNAc-6S (Delta Di-UA2S)				2A	Galα1-3Galβ1-4Galα1-3Gal				5G	Manα1-6(Manα1-3)Man				4A	GlcNAcβ1-4GlcNAc			

13 E	ΔUA-GlcNAc (Delta Di-HA)				2B	Galβ1-6Gal				5H	Manα1-6(Manα1-3)Manα1-6(Manα1-3)Man				4B	GlcNAcβ1-4GlcNAcβ1-4GlcNAc			
14 M	ΔUA→2S-GlcN-6S				2C	GalNAcβ1-3Gal				**Complex N-glycans**					4C	GlcNAcβ1-4GlcNAcβ1-4GlcNAcβ1-4GlcNAc			
14 N	ΔUA→GlcN-6S				2D	GalNAcβ1-4Gal				627	(Sia2-6A-GN-M)$_2$-3,6-M-GN-GNβ-sp4				4D	GlcNAcβ1-4GlcNAcβ1-4GlcNAcβ1-4GlcNAcβ1-4GlcNAcβ1-4GlcNAc			
14 O	ΔUA→2S-GlcN				2E	Galα1-4Galβ1-4GlcNAc				19 A	Galβ1-4GlcNAcβ1-2Manα1-3(Galβ1-4GlcNAcβ1-2Manα1-6Man)β1-4GlcNAcβ1-4(Fucα1-6)GlcNAc				4E	Bacterial cell wall muramyl discaccharide			
14P	ΔUA→GlcN				2F	GalNAcα1-3Galβ1-4Glc				19 B	Galβ1-4GlcNAcβ1-2(Galβ1-4GlcNAcβ1-4)Manα1-3(Galβ1-4GlcNAcβ1-2(Galβ1-4GlcNAcβ1-6)Manα1-6Man)β1-4GlcNAcβ1-4GlcNAc				4F	GlcNAcβ1-4GlcNAcβ1-4GlcNAcβ1-4GlcNAcβ1-4GlcNAc			
	**High molecular weight Carageenan and Glycoaminoglycans (GAGS)**				2G	Galβ1-3GlcNAcβ1-3Galβ1-4GlcNAcβ1-6(Galβ1-3GlcNAcβ1-3)Galβ1-4Glc				19 C	Neu5Acα2-6Galβ1-4GlcNAcβ1-2Manα1-3(Galβ1-4GlcNAcβ1-2Manα1-6)Manβ1-				18 G	6-O-Su-GlcNAc			

											4GlcNAcβ 1-4GlcNAc								
625	(GlcAβ1-4GlcNAcβ1-3)$_8$-NH$_2$-ol				2H	Galβ1-3GlcNAcβ 1-3Galβ1-4GlcNAcβ 1-3Galβ1-4Glc				19 D	Neu5Acα2-6Galβ1-4GlcNAcβ 1-2Manα1-3(Neu5Acα 2-6Galβ1-4GlcNAcβ 1-2Manα1-6)Manβ1-4GlcNAcβ 1-4GlcNAc				18 H	GlcNAc			
13F	(GlcAβ1-3Glc*N*Acβ1-4)n (n=4)				18 B	Galβ1-3GalNAcβ 1-3Galα1-4Galβ1-4Glc				19E	Galβ1-4GlcNAcβ 1-2Manα1-3(Galβ1-4GlcNAcβ 1-2Manα1-6)Manβ1-4GlcNAcβ 1-4GlcNAc								
13 G	(GlcAβ1-3Glc*N*Acβ1-4)n (n=8)				18 C	Galβ1-3GalNAcβ 1-3Gal				19F	Neu5Acα2-6Galβ1-4GlcNAcβ 1-2Manα1-3(Neu5Acα 2-6Galβ1-4GlcNAcβ 1-2Manα1-6)Manβ1-4GlcNAcβ 1-4(Fucα1-6)GlcNAc								
13 H	(GlcAβ1-3Glc*N*Acβ1-4)n (n=10)				18L	Galβ1-4Glc				19 G	Neu5Acα2-6Galβ1-4GlcNAcβ 1-2(Neu5Acα 2-6Galβ1-4GlcNAcβ 1-4)Manα1-3(Neu5Acα 2-6Galβ1-4GlcNAcβ								

											1-2Manα1-6)Manβ1-4GlcNAcβ1-4GlcNAc			
13I	(GlcAβ1-3Glc*N*Acβ1-4)n (n=12)				18 M	Galβ1-4Gal				19 H	GlcNAcβ1-2(GlcNAcβ1-4)Manα1-3(GlcNAcβ1-2Manα1-6)GlcNAcβ1-4Manβ1-4GlcNAcβ1-4GlcNAc			
13J	(GlcA/IdoAα/β1-4Glc*N*Acα1-4)n (n=200)				18 N	Galβ1-6Gal								
13 K	(GlcA/IdoAβ1-3(±4/6S)Gal*N*Acβ1-4)n (n<250)													
13 L	((±2S)GlcA/IdoAα/b 1-3(±4S)Gal*N*Acβ1-4)n (n<250)													
13 M	(GlcA/IdoAβ1-3(±6S)Gal*N*Acβ1-4)n (n<250)													
13 N	HA - 4 10mM													
130	HA - 6 10mM													
13P	HA - 8 9.7mM													
14 A	HA 10 7.83mM													
14 B	HA-12 6.5mM													
14 C	HA-14 5.6mM													
14 D	HA-16 4.9mM													
14 E	HA 30000 da 2.5mg/ml													
14F	HA 107000 da 2.5mg/ml													

14 G	HA 190000 da 2.5 mg/ml			
14 H	HA 220000 da 2.5 mg/ml			
14I	HA 1600000 da 2.5 mg/ml			
14J	Heparin sulfate 5 mg/ml			
14 K	β1-3Glucan			

Supplementary table 1. Heat map comparisons of glycan binding by UTI89 (WT), UTI89Δ*ytfB* and recombinant purified YtfB protein. Red indicate binding from three independent experiments, whilst white indicates not binding detected.

#	Spacer form of saccharide	Common name	Short name	Molecular weight
	**MONOSACCHARIDES**			
1	Fucα-sp3	L-α-Fuc	aF	221.3
2	Galα-sp3	α-Gal	aA	237.3
3	Galβ-sp3	β-Gal	bA	237.3
4	GalNAcα-sp0	TnSer	TnSer	308.3
5	GalNAcα-sp3	T_n	Tn	278.3
6	GalNAcβ-sp3	β-GalNAc	bAN	278.3
7	Glcα-sp3	α-Glc	aG	237.3
9	Glcβ-sp3	β-Glc	bG	237.3
10	GlcNAcβ-sp3	β-GlcNAc	GN	278.3
14	GlcN(Gc)β-sp4	β-GlcN(Gc)	bGN(Gc)	293.3
15	$HOCH_2(HOCH)_4CH_2NH_2$	aminoglucitol	glucitol	181.2
16	Manα-sp3	α-Man	aM	273.3
18	Manβ-sp4	β-Man	bM	236.2
19	ManNAcβ-sp4	β-ManAc	bMN	277.3
20	Rhaα-sp3	L-α-Rha	aR	221.3
22	GlcNAcβ-sp4	β-GlcNAc	GN-Gly	277.3
37	3-O-Su-Galβ-sp3	3-O-Su-β-Gal	bA3Su	317.3
38	3-O-Su-GalNAcα-sp3	3-O-Su-β-GalNac	bAN3Su	358.4
43	6-O-Su-GlcNAcβ-sp3	6-O-Su-β-GlcNAc	GN6su	358.4
44	GlcAα-sp3	α-glucoronic acid	aGU	251.2
45	GlcAβ-sp3	β-glucoronic acid	bGU	251.2
46	6-H_2PO_3Glcβ-sp4	β-Glc6P	G6P	316.2
47	6-H_2PO_3Manα-sp3	α-Man6P	M6P	339.2
48	Neu5Acα-sp3	α-Neu5Ac	Sia	366.4

49	Neu5Acα-sp9	α-Neu5AcBn	Sia-Bn	471.5
52	Neu5Gcα-sp3	α-Neu5Gc	aNeuGc	382.4
54	9-NAc-Neu5Acα-sp3	9-Nac-α-Neu5Ac	9NAcSia	407.4
55	3-O-Su-GlcNAcβ-sp3	3-O-Su-β-GlcNAc	GN3Su	358.4
	**DISACCHARIDES**			
71	Fucα1-2Galβ-sp3	H_{di}	Hdi	383.4
72	Fucα1-3GlcNAcβ-sp3		Fa3GN	424.5
73	Fucα1-4GlcNAcβ-sp3	Le	LeC	424.5
75	Galα1-2Galβ-sp3		Aa2A	399.4
76	Galα1-3Galβ-sp3	B_{di}	Bdi	399.4
77	Galα1-3GalNAcβ-sp3	$T_{\alpha\beta}$	Tab	440.5
78	Galα1-3GalNAcα-sp3	$T_{\alpha\alpha}$	Taa	440.5
80	Galα1-3GlcNAcβ-sp3		Aa3GN	440.5
81	Galα1-4GlcNAcβ-sp3	α-LacNAc	aLN	440.5
83	Galα1-6Glcβ-sp4	Melibiose	Aa3G	398.4
84	Galβ1-2Galβ-sp3		Ab2A	399.4
85	Galβ1-3GlcNaAcβ-sp3	Le^c	LeC	440.5
87	Galβ1-3Galβ-sp3		Ab3A	399.4
88	Galβ1-3GalNAcβ-sp3	$T_{\beta\beta}$	Tbb	440.5
89	Galβ1-3GalNAcα-sp3	TF	TF	440.5
93	Galβ1-4Glcβ-sp4	Lac	Lac-Gly	398.4
94	Galβ1-4Galβ-sp4		Ab4A	398.4
97	Galβ1-4GlcNAcβ-sp3	LacNAc	LN	440.5
100	Galβ1-6Galβ-sp4		Ab6A	398.4
101	GalNAcα1-3GalNAcβ-sp3	Fs-2	Fs-2	481.5
102	GalNAcα1-3Galβ-sp3	A_{di}	Adi	440.5

103	GalNAcα1-3GalNAcα-sp3	core 5	core 5	481.5
104	GalNAcβ1-3Galβ-sp3		ANb3A	440.5
106	GalNAcβ1-4GlcNAcβ-sp3	LacdiNAc	LacdiNAc	481.5
110	Glcα1-4Glcβ-sp3	maltose	Malt2	399.4
111	Glcβ1-4Glcβ-sp4	cellobiose	cello	398.4
112	Glcβ1-6Glcβ-sp4	gentiobiose	gent	398.4
113	GlcNAcβ1-3GalNAcα-sp3	core 3	core 3	481.5
114	GlcNAcβ1-3Manβ-sp4		GN3M	439.4
115	GlcNAcβ1-4GlcNAcβ-Asn	chitobiose-Asn	Ch2-Asn	538.5
117	GlcNAcβ1-4GlcNAcβ-sp4	chitobiose	Ch2-Gly	480.5
118	GlcNAcβ1-6GalNAcα-sp3	core 6	core 6	481.5
119	Manα1-2Manβ-sp4		Ma2Mb	398.4
120	Manα1-3Manβ-sp4		Ma3M	398.4
121	Manα1-4Manβ-sp4		Ma4M	398.4
122	Manα1-6Manβ-sp4		Ma6M	398.4
123	Manβ1-4GlcNAcβ-sp4		Mb4GN	439.4
124	Manα1-2Manα-sp4		Ma2Ma	398.4
145	Galβ1-3(6-O-Su)GlcNAcβ-sp3	6-O-Su-Le^{c}	LeC6Su	520.5
146	Galβ1-4(6-O-Su)Glcβ-sp2	6-O-Su-Lac	Lac6Su	465.5
147	Galβ1-4(6-O-Su)GlcNAcβ-sp3	6-O-Su-LacNAc	LN6Su	520.5
149	GlcNAcβ1-4(6-O-Su)GlcNAcβ-sp2	6-O-Su-chitobiose	Ch2-6Su	547.5
150	3-O-Su-Galβ1-3GalNAcα-sp3	3`-O-Su-TF	TF3`Su	520.5
151	6-O-Su-Galβ1-3GalNAcα-sp3	6`-O-Su-TF	TF6`Su	520.5
152	3-O-Su-Galβ1-4Glcβ-sp2	SM3	Lac3`Su	465.3
153	6-O-Su-Galβ1-4Glcβ-sp2	6`-O-Su-Lac	Lac6`Su	465.3
155	3-O-Su-Galβ1-3GlcNAcβ-sp3	3`-O-Su-Le^{c}	LeC3`Su	520.5

157	3-O-Su-Galβ1-4GlcNAcβ-sp3	3`-O-Su-LacNAc	LN3`Su	520.5
159	4-O-Su-Galβ1-4GlcNAcβ-sp3	4`-O-Su-LacNAc	LN4`Su	520.5
161	6-O-Su-Galβ1-3GlcNAcβ-sp3	6`-O-Su-Le^c	LeC6`Su	520.5
163	6-O-Su-Galβ1-4GlcNAcβ-sp3	6`-O-Su-LacNAc	LN6`Su	520.5
164	GlcAβ1-3GlcNAcβ-sp3		GUb3GN	454.4
165	GlcAβ1-3Galβ-sp3		GUb3A	413.4
166	GlcAβ1-6Galβ-sp3		GUb6A	413.4
167	GlcNAcβ1-4-[HOOC(CH_3)CH]-3-O-GlcNAcβ-sp4	GlcNAc-Mur	GN-Mur	522.5
168	GlcNAcβ1--[HOOC(CH_3)CH]-3-O-GlcNAcβ-L-alanyl-D-i-glutaminyl-L-lysine	GMDP-Lys	GMDPLys	823.9
169	Neu5Acα2-3Galβ-sp3	GM4	GM4	528.5
170	Neu5Acα2-6Galβ-sp3		Sia6A	528.5
171	Neu5Acα2-3GalNAcα-sp3	3-SiaT_n	3-SiaTn	569.6
172	Neu5Acα2-6GalNAcα-sp3	SiaT_n	SiaTn	569.6
174	Neu5Gcα2-6GalNAcα-sp3	NeuGc-T_n	Neu5GCTn	585.6
176	3-O-Su-Galβ1-4(6-O-Su)Glcβ-sp2	3`,6-di-O-Su-Lac	Lac3`,6Su2	567.5 (Na+)
177	3-O-Su-Galβ1-4(6-O-Su)GlcNAcβ-sp2	3`,6-di-O-Su-LacNAc	LN3`6Su2	622.6 (Na+)
178	6-O-Su-Galβ1-4(6-O-Su)Glcβ-sp2	6,6`-di-O-Su-Lac	Lac6,6`Su2	567.5 (Na+)
179	6-O-Su-Galβ1-3(6-O-Su)GlcNAcβ-sp2	6,6`-di-O-Su-Le^c	LeC6,6`Su2	608.5 (Na+)
180	6-O-Su-Galβ1-4(6-O-Su)GlcNAcβ-sp2	6,6`-di-O-Su-LacNAc	LN66`Su2	608.5 (Na+)
181	3,4-O-Su_2-Galβ1-4GlcNAcβ-sp3	3`,4`-di-O-Su-LacNAc	LN3`4`Su2	622.6 (Na+)
182	3,6-O-Su_2-Galβ1-4GlcNAcβ-sp2	3`,6`-di-O-Su-LacNAc	LN3`6`Su2	608.5 (Na+)
183	4,6-O-Su_2-Galβ1-4GlcNAcβ-sp2	4`,6`-di-O-Su-LacNAc	LN4`6`Su2	608.5 (Na+)
184	4,6-O-Su_2-Galβ1-4GlcNAcβ-sp3	4`,6`-di-O-Su-LacNAc	LN4`6`Su2-C3	622.6 (Na+)
186	Neu5Acα2-8Neu5Acα2-sp3	[32]	[32]	679.6 (Na+)
189	3,6-O-Su_2-Galβ1-4(6-O-Su)GlcNAcβ-sp2	3`,6,6`-tri-O-Su-LacNAc	LN3`66`Su3	710.6 (Na+)
192	GalNAcβ1-4(6-O-Su)GlcNAcβ-sp3	6-O-Su-LacdiNAc	LacdiNAc6Su	561.5

193	3-O-Su-GalNAcβ1-4GlcNAcβ-sp3	3`-O-Su-LacdiNAc	LacdiNAc3`Su	561.5
194	6-O-Su-GalNAcβ1-4GlcNAcβ-sp3	6`-O-Su-LacdiNAc	LacdiNAc6`Su	561.5
195	6-O-Su-GalNAcβ1-4-(3-O-Su)GlcNAcβ-sp3	6`-Su-3-O-Ac-LacdiNAc	3Ac-LacdiNAc6`Su	603.5
196	3-O-Su-GalNAcβ1-4(3-O-Su)-GlcNAcβ-sp3	3,3`-O-Su_2-LacdiNAc	LacdiNAc3,3`Su2	663.5(Na+)
197	3,6-O-Su_2-GalNAcβ1-4GlcNAcβ-sp3	3`,6`-Su_2-LacdiNAc	LacdiNAc3`,6`Su2	663.5(Na+)
198	4,6-O-Su_2-GalNAcβ1-4GlcNAcβ-sp3	4`,6`-Su_2-LacdiNAc	LacdiNAc4`,6`Su2	663.5(Na+)
199	4,6-O-Su_2-GalNAcβ1-4-(3-O-Ac)GlcNAcβ-sp3	4`,6`-Su_2-3-O-Ac-LacdiNAc	3Ac-LacdiNAc4`,6`Su2	705.5(Na+)
200	4-O-Su-GalNAcβ1-4GlcNAcβ-sp3	4`-O-Su-LacdiNAc	LacdiNAc4`Su	561.5
201	3,4-O-Su_2-Galβ1-4GlcNAcβ-sp3	3`,4`-Su_2-LacdiNAc	LacdiNAc3`,4`Su2	663.5(Na+)
202	6-O-Su-GalNAcβ1-4(6-O-Su)GlcNAcβ-sp3	6,6`-O-Su-LacdiNAc	LacdiNAc6,6`Su2	663.5(Na+)
203	Galβ1-4(6-O-Su)GlcNAcβ-sp2	6-O-Su-LacNAc	LN6Su	506.5
204	4-O-Su-GalNAcβ1-4GlcNAcβ-sp2	4`-O-Su-LacdiNAc	LacdiNAc4`Su-C2	547.5
205	Neu5Acα2-6GalNAcβ-sp3		6SiaANb	569.6
206	Neu5Gcα2-3Gal-sp3	NeuGcα3Gal	Neu5Gc3A	544.5
	**TRISACCHARIDES**			
215	Fucα1-2Galβ1-3GlcNAcβ-sp3	$Le^{d,}$ H (type 1)	LeD	586.6
216	Fucα1-2Galβ1-4GlcNAcβ-sp3	H (type 2)	Htype2	586.6
217	Fucα1-2Galβ1-3GalNAcα-sp3	H (type 3)	Htype3	586.6
219	Fucα1-2Galβ1-4Glcβ-sp4	H (type 6	Htype6	544.5
220	Galα1-3Galβ1-4Glcβ-sp2		Aa3`Lac-C2	547.5
222	Galα1-3Galβ1-4GlcNAcβ-sp3	Galili [4]	Galili3	602.6
224	Galα1-4Galβ1-4Glcβ-sp3	P^k, Gb3, $GbOse_3$	Pk	561.5
225	Galα1-4Galβ1-4GlcNAc-sp2	P_1	P1	588.6
226	Fucα1-2(Galα1-3)Galβ-sp3	B_{tri}	Btri	545.5
228	Galβ1-2Galα1-4GlcNAcβ-sp4		Ab2aLN	601.6
229	Galβ1-3Galβ1-4GlcNAcβ-sp4		Ab3`LN	601.6

231	Galβ1-4GlcNAcβ1-3GalNAcα-sp3		LN3Tn	643.6
232	Galβ1-4GlcNAcβ1-6GalNAcα-sp3		LN6Tn	643.6
233	Galβ1-3(Fucα1-4)GlcNAcβ-sp3	Le^a	LeA	586.6
234	Fucα1-3(Galβ1-4)GlcNAcβ-sp3	Le^x	LeX	586.6
235	Fucα1-2(GalNAcα1-3)Galβ-sp3	A_{tri}	Atri	586.6
238	GalNAcβ1-4Galβ1-4Glcβ-sp3	GA_2, $GgOse_3$	GA2	602.6
240	(Glcα1-4)$_3$β-sp4	maltotriose	(Ga4)3b	560.5
241	(Glcα1-6)$_3$β-sp4	isomaltotriose	(Ga6)3b	560.5
246	GlcNAcβ1-2Galβ1-3GalNAcα-sp3		GN2`TF	643.6
247	GlcNAcβ1-3Galβ1-3GalNAcα-sp3		GN3`TF	643.6
248	GlcNAcβ1-3Galβ1-4Glcβ-sp2		GN3`Lac	588.6
250	GlcNAcβ1-3Galβ1-4GlcNAcβ-sp3		GN3`LN	643.6
251	GlcNAcβ1-4Galβ1-4GlcNAcβ-sp2		GN4`LN	629.6
252	GlcNAcβ1-4GlcNAcβ1-4GlcNAcβ-sp4	chitotriose	Ch3	683.6
253	GlcNAcβ1-6Galβ1-4GlcNAcβ-sp2		GN6`LN	629.6
254	Galβ1-3(GlcNAcβ1-6)GalNAcα-sp3	core 2	core 2	643.6
255	GlcNAcβ1-3(GlcNAcβ1-6)GalNAcα-sp3	core 4	core 4	684.7
258	Manα1-3(Manα1-6)Manβ-sp4	Man_3	(Ma)3b	560.5
262	Galβ1-3GalNAcβ1-3Gal-sp4	$T_{\beta\beta}$-Gal	Tbb-A	601.6
264	Galβ1-4Galβ1-4GlcNAc-sp3		Ab4`LN	602.6
287	3-O-Su-Galβ1-3(Fucα1-4)GlcNAcβ-sp3	Su-Le^a	3`SuLeA	666.7
288	Fucα1-3(3-O-Su-Galβ1-4)GlcNAcβ-sp3	Su-Le^x	3`SuLeX	666.7
289	Galα1-3(Neu5Acα2-6)GalNAcα-sp3	6-SiaTF	6SiaTF	731.7
290	Galβ1-3(Neu5Acα2-6) GalNAcα-sp3		$A3a^3Tn$	731.7
292	Neu5Acα2-3Galβ1-3GalNAcα-sp3	3`-Sia-TF	Sia3`TF	731.7
293	Neu5Acα2-3Galβ1-4Glcβ-sp3	3`SL	3`SL	690.7

294	Neu5Acα2-3Galβ1-4Glcβ-sp4	3`SL	3`SL-Gly	689.6
295	Neu5Acα2-6Galβ1-4Glcβ-sp2	6`SL	6`SL-C2	676.6
298	Neu5Acα2-3Galβ1-4GlcNAcβ-sp3	3`SLN	3`SLN	731.7
299	Neu5Acα2-3Galβ1-3GlcNAcβ-sp3	3`-SiaLe^{c}	3`SiaLeC	731.7
300	Neu5Acα2-6Galβ1-4GlcNAcβ-sp3	6`SLN	6`SLN	731.7
303	Neu5Gcα2-3Galβ1-4GlcNAcβ-sp3	3`SLN (Gc)	3`SLN(Gc)	747.7
304	Neu5Gcα2-6Galβ1-4GlcNAcβ-sp3	6`SLN (Gc)	6`SLN(Gc)	747.7
306	9-NAc-Neu5Acα2-6Galβ1-4GlcNAcβ-sp3		9NAc-6`SLN	788.8
315	Neu5Acα2-3Galβ1-4-(6-O-Su)GlcNAcβ-sp3	6-Su-3`SLN	3`SLN6Su	833.8 (Na+)
317	Neu5Acα2-3Galβ1-3-(6-O-Su)GalNAcβ-sp3	6-Su-3`SiaTF	3`SiaTF6Su	833.8 (Na+)
318	Neu5Acα2-6Galβ1-4-(6-O-Su)GlcNAcβ-sp3	6-Su-6`SLN	6`SLN6Su	833.8 (Na+)
319	Neu5Acα2-3-(6-O-Su)Galβ1-4GlcNAcβ-sp3	6`-Su-3`SLN	3`SLN6`Su	833.8 (Na+)
321	(Neu5Acα2-8)$_3$-sp3	[33]	[33]	992.9 (Na+)
323	Neu5Acα2-6Galβ1-3GlcNAc-sp3	6`-SiaLe^{c}	6`SiaLeC	731.7
324	Neu5Acα2-6Galβ1-3(6-O-Su)GlcNAc-sp3	6Su-6`-SiaLe^{c}	6`SiaLeC6Su	883.8 (Na+)
331	Neu5Gcα2-3Galβ1-3GlcNAcβ-sp3	3`SiaLe^{c} (Gc)	3`SiaLeC(GC)	747.7
	**TETRASACCHARIDES**			
359	Fucα1-2(Galα1-3)Galβ1-3GlcNAcβ-sp3	B (type 1)	Btype1	748.7
360	Fucα1-2(Galα1-3)Galβ1-4GlcNAcβ-sp3	B (type 2)	Btype2	748.7
362	Fucα1-2(Galα1-3)Galβ1-3GalNAcα-sp3	B (type 3)	Btype3	748.7
363	Fucα1-2(Galα1-3)Galβ1-3GalNAcβ-sp3	B (type 4)	Btype4	748.7
364	Fucα1-3(Galα1-3Galβ1-4)GlcNAcβ-sp3	αGalLe^{x}	aGalLeX	748.7
366	Fucα1-2(GalNAcα1-3)Galβ1-3GlcNAcβ-sp3	A (type 1)	Atype1	789.8
368	Fucα1-2(GalNAcα1-3)Galβ1-4GlcNAcβ-sp3	A (type 2)	Atype2	789.8
371	Fucα1-2Galβ1-3(Fucα1-4)GlcNAcβ-sp3	Le^{b}	LeB	732.7
372	Fucα1-3(Fucα1-2Galβ1-4)GlcNAcβ-sp3	Le^{y}	LeY	732.7

373	Galα1-3Galβ1-4GlcNAcβ1-3Galβ-sp3	Galili (tetra)	Galili4	764.7
375	Galα1-4GlcNAcβ1-3Galβ1-4GlcNAcβ-sp3		aLN3`LN	805.8
376	Galβ1-3GlcNAcβ1-3Galβ1-4Glcβ-sp4	LNT	LNT	763.7
377	Galβ1-3GlcNAcβ1-3Galβ1-3GlcNAcβ-sp2		LeCb3`LeC	791.8
378	Galβ1-3GlcNAcα1-3Galβ1-4GlcNAcβ-sp3		LeCa3`LN	805.8
379	Galβ1-3GlcNAcβ1-3Galβ1-4GlcNAcβ-sp3		LeCb3`LN	805.8
380	Galβ1-3GlcNAcα1-6Galβ1-4GlcNAcβ-sp2		LeCa6`LN	791.8
381	Galβ1-3GlcNAcβ1-6Galβ1-4GlcNAcβ-sp2		LeCb6`LN	791.8
382	Galβ1-3GalNAcβ1-4Galβ1-4Glcβ-sp3	Asialo-GM1	aGM1	764.7
383	Galβ1-4GlcNAcβ1-3Galβ1-4Glcβ-sp2	LNnT	LNnT	763.7
385	Galβ1-4GlcNAcβ1-3Galβ1-4GlcNAcβ-sp3	i	LNb3`LN	805.8
387	Galβ1-4GlcNAcβ1-6Galβ1-4GlcNAcβ-sp2		LNb6`LN	791.7
388	Galβ1-3(Galβ1-4GlcNAcβ1-6)GalNAcα-sp3		LNb6TF	805.8
389	GalNAcβ1-3Galα1-4Galβ1-4Glcβ-sp3	Gb4, P	Gb4	764.7
390	(Glcα1-4)$_4$β-sp4	maltotetraose	(Ga4)4b	722.7
391	(Glcα1-6)$_4$β-sp4	isomaltotetraose	(Ga6)4b	722.7
392	Fucα1-2(GalNAcα1-3)Galβ1-3GalNAcα-sp3	A (type 3)	A(type3)	789.8
395	GlcNAcβ1-3(GlcNAcβ1-6)Galβ1-4GlcNAcβ-sp3	Tk	Tk	832.8
401	Galβ1-3GlcNAcβ1-3Galβ1-3GlcNAcβ-sp3	Le^{c}3Le^c	Le^{c}3Le^c	805.8
419	3-O-SuGalβ1-4GlcNAcβ1-3Galβ1-4GlcNAcβ-sp3		(3`SuLN)3`LN	907.8
420	4-O-SuGalβ1-4GlcNAcβ1-3Galβ1-4GlcNAcβ-sp3		(4`SuLN)3`LN	907.8
421	Neu5Acα2-3(GalNAcβ1-4)Galβ1-4Glcβ-sp2	GM2	GM2	879.8
422	Neu5Acα2-3Galβ1-4GlcNAcβ1-3Galβ-sp3		3`SLNb3A	893.9
423	Fucα1-3(Neu5Acα2-3Galβ1-4)GlcNAcβ-sp3	SiaLe^x	SiaLeX	877.9
426	Neu5Acα2-3Galβ1-3(Fucα1-4)GlcNAcβ-sp3	SiaLe^a	SiaLeA	877.9
428	Fucα1-3(Neu5Acα2-3Galβ1-4)6-O-Su-GlcNAcβ-sp3		SiaLeX6Su	979.9

429	Fucα1-3(Neu5Acα2-3(6-O-Su)Galβ1-4)GlcNAcβ-sp3		SiaLeX6`Su	979.9
433	Neu5Acα2-3Galβ1-3(Neu5Acα2-6)GalNAcα-sp3	Sia$_2$-TF	Sia2-3`,6TF	1044.9
434	Neu5Acα2-8Neu5Acα2-3Galβ1-4Glcβ-sp4	GD3	GC3	1002.9 (Na+)
	**PENTA-NONA SACCHARIDES**			
479	Fucα1-2Galβ1-3GlcNAcβ1-3Galβ1-4Glcβ-sp4	LNFP-I	Htype1Lac	909.9
480	Fucα1-2Galβ1-3GlcNAcβ1-3Galβ1-4GlcNAcβ-sp2	H (type1) penta	HtypeILN	937.91
481	Galα1-3Galβ1-4GlcNAcβ1-3Galβ1-4Glcβ-sp4	Galili (penta)	Galili5	925.8
483	Fucα1-3(Fucα1-2 (Galα1-3)Galβ1-4)GlcNAcβ-sp3	BLe^y	BLeY	894.9
488	Galβ1-4GlcNAcβ1-3(Galβ1-4GlcNAcβ1-6)GalNAcα-sp3		LN2-3,6Tn	1008.9
489	Galβ1-4GlcNAcβ1-3(GlcNAcβ1-6)Galβ1-4GlcNAc-sp2		LN3`(GN6`)LN	994.9
490	GlcNAcβ1-3(Galβ1-4GlcNAcβ1-6)Galβ1-4GlcNAcβ-sp2		LN6`(GN3`)LN	994.9
492	(Glcα1-6)$_5$β-sp4	isomaltopentaose	(Ga6)5b	884.8
493	(GlcNAcβ1-4)$_5$β-sp4	chitopentaose	Ch5	1090
495	Manα1-3(Manα1-3(Manα1-6)Manα1-6)Manβ-sp4	Man5	(Ma)5b	966.9
496	Fucα1-2Galβ1-3(Fucα1-4)GlcNAcβ1-3Galβ1-4Glcβ-sp4	Le^b-Lac	LeBLac	1056
497	Fucα1-3(Fucα1-2Galβ1-4)GlcNAcβ1-3Galβ1-4Glcβ-sp4	Le^y-Lac	LeYLac	1056
498	(Galβ1-4GlcNAcβ1-3)$_3$-sp3	(LN)$_3$	(LNb3`)3	1171.1
499	Galβ1-4GlcNAcβ1-3(Galβ1-4GlcNAcβ1-6)Galβ1-4GlcNAc-sp2	I	LN2-3`,6`LN	1157.1
501	Galβ1-3GalNAcβ1-3Galα1-4Galβ1-4Glcβ-sp4	Gb5	Gb5	925.8
502	(Glcα1-6)$_6$β-sp4	maltohexaose	(Ga6)6b	1046.9
503	(GlcNAcβ1-4)$_6$β-sp4	chitohexaose	Ch6	1293.3
504	(A-GN-M)$_2$-3,6-M-GN-GNβ-sp4	9-OS	9-OS	1697.6
505	(GN-M)$_2$-3,6-M-GN-GNβ-sp4	7-OS	7-OS	1373.3
527	Neu5Acα2-3Galβ1-4GlcNAcβ1-3Galβ1-4GlcNAcβ-sp2	3`SLN-LacNAc	3`SLN-LN	1083
528	Fucα1-3(Neu5Acα2-3 Galβ1-4)GlcNAcβ1-3Galβ-sp3	$SiaLe^x$-3Gal	SiaLeX3A	1040
529	Neu5Acα2-6(Galβ1-3)GlcNAcβ1-3Galβ1-4Glcβ-sp4	LSTb	LSTb	1055

531	GalNAcβ1-4(Neu5Acα2-8Neu5Acα2-3)Galβ1-4Glc-sp2	GD2	GD2-C2	1193.1 (Na+)
532	Neu5Acα2-8Neu5Acα2-8Neu5Acα2-3Galβ1-4Glc-sp2	GT3	GT3	1303.1 (2Na+)
533	(Neu5Acα2-8)2Neu5Acα2-3(GalNAcβ1-4)Galβ1-4Glc-sp2	GT2	GT2	1506.3 (2Na+)
534	Neu5Acα2-3Galβ1-4GlcNAcβ1-3Galβ1-4GlcNAcβ-sp3	6`SLN-LacNAc	6`SLN-LN	1097.1
536	Neu5Acα2-3Galβ1-3GlcNAcβ1-3Galβ1-4Glcβ-sp4	LSTa	LSTa	1055
537	Neu5Acα2-3Galβ1-4GlcNAcβ1-3Galβ1-4Glcβ-sp4	LSTd	LSTd	1055
538	Le^x1-6'(Le^c1-3')Lac-sp4	MFLNH III	MFLNH III	1389.2
539	LacNAc1-6'(Le^d1-3')Lac-sp4	MFLNH I	MFLNH I	1389.2
540	Le^x1-6'(6'SLN1-3')Lac-sp4	MSMFLNnH	MSMFLNnH	1566.5
541	Le^x1-6'(Le^d1-3')Lac-sp4	DFLNH (a)	DFLNH (a)	1535.4
542	Le^cLe^x1-6'(Le^c1-3')Lac-sp4	MF(1-3)iLNO	MF(1-3)iLNO	1754.6
543	Le^x1-6'(Le^b1-3')Lac-sp4	TFLNH	TFLNH	1681.5
	**HIGER OLIGOSACCHARIDES**			
625	$(GlcA\beta1\text{-}4GlcNAc\beta1\text{-}3)_8$-$NH_2$-ol	hyaluroninc acid	HyalU-ol	3207.5
627	$(Sia2\text{-}6A\text{-}GN\text{-}M)_2$-3,6-M-GN-GNβ-sp4	11-OS, YDS	11-OS	2302.1 (Na+)
	**Terminal galactose**			
1A	Galβ1-3GlcNAc	Lacto-N-Biose I		383.35
1B	Galβ1-4GlcNAc	N-Acetyllactosamine		383.35
1C	Galβ1-4Gal	β-1-4-galactosyl-galactose		342.3
1D	Galβ1-6GlcNAc	β-1-6 Galactosyl-N-acetyl glucosamine		383.35
1E	Galβ1-3GalNAc	β-1-3 Galactosyl-N-acetyl galactosamine		383.35
1F	Galβ1-3GalNAcβ1-4Galβ1-4Glc	asialo GM1		707.63
1G	Galβ1-3GlcNAcβ1-3Galβ1-4Glc	Lacto-N-tetraose		707.63
1H	Galβ1-4GlcNAcβ1-3Galβ1-4Glc	Lacto-N-neotetraose		707.63
1I	Galβ1-4GlcNAcβ1-6(Galβ1-4GlcNAcβ1-3)Galβ1-4Glc	Lacto-N-neohexaose		1072.96
1J	Galβ1-4GlcNAcβ1-6(Galβ1-3GlcNAcβ1-3)Galβ1-4Glc	Lacto-N-hexaose		1072.96

1K	Galα1-4Galβ1-4Glc	Globotriose	504.44
1L	GalNAcα1-O-Ser	Tn Antigen GalNAcα1-O-Ser	308.29
1M	Galβ1-3GalNAcα1-O-Ser	Galactosyl-Tn Antigen	470.43
1N	Galα1-3Gal	α1-3 Galactobiose	342.24
1O	Galα1-3Galβ1-4GlcNAc	Linear B-2 Trisaccharide	545.49
1P	Galα1-3Galβ1-4Glc	Linear B-6 Trisaccharide	504.44
2A	Galα1-3Galβ1-4Galα1-3Gal	α1-3, β1-4,α1-3 Galactotetraose	666.58
2B	Galβ1-6Gal	beta1-6galactobiose	342.3
2C	GalNAcβ1-3Gal	terminal disaccharide of Globotriose	383.35
2D	GalNAcβ1-4Gal	receptor for P.aureginosa	383.35
2E	Galα1-4Galβ1-4GlcNAc	P1 antigen	545.49
2F	GalNAcα1-3Galβ1-4Glc	a-D-N-acetylgalactosamine 1-3Galβ1-4Glc	545.49
2G	Galβ1-3GlcNAcβ1-3Galβ1-4GlcNAcβ1-6(Galβ1-3GlcNAcβ1-3)Galβ1-4Glc	iso-Lacto-N-octaose (iLNO)	1438.3
2H	Galβ1-3GlcNAcβ1-3Galβ1-4GlcNAcβ1-3Galβ1-4Glc	para-Lacto-N-hexaose (pLNH)	1072.96
18B	Galβ1-3GalNAcβ1-3Galα1-4Galβ1-4Glc	Globopentaose	869.76
18C	Galβ1-3GalNAcβ1-3Gal	Core type 4/Gb5 triose structure	545.48
18L	Galβ1-4Glc	4-O-(B-D-galactopyranosyl)-B-D-glucose	342.3
18M	Galβ1-4Gal	4-O-(B-D-galactopyranosyl)-B-D-galactose	360.3
18N	Galβ1-6Gal	6-O-(B-D-galactopyranosyl)-B-D-galactose	342.3
	**Terminal GlcNAc**		
4A	GlcNAcβ1-4GlcNAc	N,N'-Diacetyl chitobiose	424.4
4B	GlcNAcβ1-4GlcNAcβ1-4GlcNAc	N,N',N''-Triacetyl chitotriose	627.59
4C	GlcNAcβ1-4GlcNAcβ1-4GlcNAcβ1-4GlcNAc	N,N',N'',N'''-Tetraacetyl chitotetraose	830.79
4D	GlcNAcβ1-4GlcNAcβ1-4GlcNAcβ1-4GlcNAcβ1-4GlcNAcβ1-4GlcNAc	N,N',N'',N''',N'''',N'''''-Hexaacetyl chitohexaose	1237.17
4E	GlcNAcβ1-4MurNAc	Bacterial cell wall muramyl discaccharide	496.46

4F	GlcNAcβ1-4GlcNAcβ1-4GlcNAcβ1-4GlcNAcβ1-4GlcNAc	Pentacetyl chitopentaose	1033.98
18G	6-O-Su-GlcNAc	N-Acetyl-D-Glucosamine 6-O-sulfate	323.25
18H	GlcNAc	N-Acetyl-D-Glucosamine	221.21
	**Mannosyl containing glycans**		
5A	GlcNAcβ1-2Man	β1-2 N-Acetylglucosamine-mannose	383.35
5B	GlcNAcβ1-2Manα1-6(GlcNAcβ1-2Manα1-3)Man	Bianntennary N-linked core pentasaccharide	910.8
5C	Manα1-2Man	α1-2-Mannobiose	342.3
5D	Manα1-3Man	α1-3-Mannobiose	342.3
5E	Manα1-4Man	α1-4-Mannobiose	342.3
5F	Manα1-6Man	α1-6-Mannobiose	342.3
5G	Manα1-6(Manα1-3)Man	α1-3,α1-6-Mannobiose	504.44
5H	Manα1-6(Manα1-3)Manα1-6(Manα1-3)Man	α1-3,α1-3,α1-6-Mannopentaose	828.72
	**Fucosylated glycans**		
7A	Fucα1-2Galβ1-3GlcNAcβ1-3Galβ1-4Glc	Lacto-N-fucopentaose I	853.77
7B	Galβ1-3(Fucα1-4)GlcNAcβ1-3Galβ1-4Glc	Lacto-N-fucopentaose II	853.77
7C	Galβ1-4(Fucα1-3)GlcNAcβ1-3Galβ1-4Glc	Lacto-N-fucopentaose III	853.77
7D	Fucα1-2Galβ1-3(Fucα1-4)GlcNAcβ1-3Galβ1-4Glc	Lacto-N-difucohexaose I	999.91
7E	Galβ1-3(Fucα1-4)GlcNAcβ1-3Galβ1-4(Fucα1-3)Glc	Lacto-N-difucohexaose II	999.91
7F	Fucα1-2Gal	H-disaccaride	326.3
7G	Fucα1-2Galβ1-4Glc	2'-Fucosyllactose	488.44
7H	Galβ1-4(Fucα1-3)Glc	3'-Fucosyllactose	488.44
7I	Galβ1-4(Fucα1-3)GlcNAc	$Lewis^{x}$	529.49
7J	Galβ1-3(Fucα1-4)GlcNAc	$Lewis^{a}$	529.49
7K	GalNAcα1-3(Fucα1-2)Gal	Blood Group A trisaccharide	529.49
7L	Fucα1-2Galβ1-4(Fucα1-3)Glc	Lactodifucotetraose (LDFT)	

7M	Galβ1-3(Fucα1-2)Gal	Blood Group B Trisaccharide	488.44
7N	Fucα1-2Galβ1-4(Fucα1-3)GlcNAc	Lewis[y]	675.63
7O	Fucα1-2Galβ1-3GlcNAc	Blood Group H Type II Trisaccharide	529.49
7P	Fucα1-2Galβ1-3(Fucα1-4)GlcNAc	Lewis[b] tetrasaccharide	675.63
8A	SO_3-3Galβ1-3(Fucα1-4)GlcNAc	Sulpho Lewis[a]	631.53
8B	SO_3-3Galβ1-4(Fucα1-3)GlcNAc	Sulpho Lewis[x]	631.53
8C	Galβ1-3GlcNAcβ1-3Galβ1-4(Fucα1-3)GlcNAcβ1-3Galβ1-4Glc	Monofucosyl-para-Lacto-N-hexaose IV	1219.1
8D	Galβ1-4(Fucα1-3)GlcNAcβ1-6(Galβ1-3GlcNAcβ1-3)Galβ1-4Glc	Monofucosyllacto-N-hexaose III	1219.1
8E	Galβ1-4(Fucα1-3)GlcNAcβ1-6(Fucα1-2Galβ1-3GlcNAcβ1-3)Galβ1-4Glc	Difucosyllacto-N-hexaose	1365.25
8F	Galβ1-4(Fucα1-3)GlcNAcβ1-6(Fucα1-2Galβ1-3(Fucα1-4)GlcNAcβ1-3)Galβ1-4Glc	Trifucosyllacto-N-hexaose	1511.39
8G	Galβ1-4GlcNAcβ1-3Galβ1-4(Fucα1-3)Glc	Lacto-N-fucopentaose VI (LNFP VI)	853.77
8H	Fucα1-2Galβ1-4(Fucα1-3)GlcNAcβ1-3Galβ1-4Glc	Lacto-N-neodifucohexaose I (LNnDFH I)	999.91
8I	Fucα1-3Galβ1-4GlcNAcβ1-3Galβ1-4(Fucα1-3)Glc	Lacto-N-neodifucohexaose II (LNnDFH II)	999.91
8J	Fucα1-2Galβ1-4(Fucα1-3)GlcNAcβ1-3(Fucα1-2)Galβ1-4Glc	Trifucosyllacto-N-neoteraose I (TFLNnTI)	1146.05
8K	Galβ1-4(Fucα1-3)GlcNAcβ1-6(Galβ1-4GlcNAcβ1-3)Galβ1-4Glc	Monofucosyllacto- N-neohexaose I (MFLNnH I)	1219.1
8L	Galβ1-4(Fucα1-3)GlcNAcβ1-6(Galβ1-4(Fucα1-3)GlcNAcβ1-3)Galβ1-4Glc	Difucosyllacto-N-neohexaose I (DFLNnH I)	1365.25
8M	Fucα1-2Galβ1-4(Fucα1-3)GlcNAcβ1-6(Galβ1-4GlcNAcβb1-3)Galβ1-4Glc	Difucosyllacto-N-neohexaose II (DFLNnH II)	1365.25
8N	Galβ1-3GlcNAcβ1-3Galβ1-4(Fucα1-3)GlcNAcβ1-6(Galβ1-3GlcNAcβ1-3)Galβ1-4Glc	Monofucosyl(1-3)-iso-lacto-N-octaose (MFiLNO)	1584.44
8O	Fucα1-2Galβ1-3GlcNAcβ1-3Galβb1-4(Fucα1-3)GlcNAcβ1-6(Galβ1-3GlcNAcβ1-3)Galβ1-4Glc	Trifucosyl(1-2,1-2,1-3)-iso-lacto-N-octaose (TFiLNO (1-2,1-2,1-3))	1876.72
8P	GalNAcα1-3(Fucα1-2)Galβ1-4GalNAc	Blood Group A tetrasaccharide	732.68
9A	Galα1-3(Fucα1-2)Galβ1-4(Fucα1-3)Glc	Blood Group B pentasaccharide	796.72
9B	Galβ1-4GlcNAcβ1-6(Fucα1-2Galβ1-3GlcNAcβ1-3)Galβ1-4Glc	Monofucosyllacto-N-hexaose I	1219.1
18D	Galα1-3(Fucα1-2)Galβ1-4Glc	Blood group B antigen tetraose type 5	650.57

18E	GalNAcα1-3(Fucα1-2)Galβ1-4(Fucα1-3)Glc	Blood group A pentasaccharide	837.77
19J	Galβ1-4(Fucα1-3)GlcNAcβ1-3Gal	Lewis^{x} tetraose	691.62
19L	Fucα1-2Galβ1-4(Fucα1-3)GlcNAcβ1-3Gal	Lewis^{y} pentaose	837.77
19M	Galβ1-3(Fucα1-4)GlcNAcβ1-3Gal	Lewis^{a} tetraose	691.62
19N	Fucα1-2Galβ1-3(Fucα1-4)GlcNAcβ1-3Gal	Lewis^{b} pentaose	837.77
20A	Fucα1-2Galβ1-3GalNAcβ1-3Gal	Blood group H antigen tetraose type 4	691.27
20B	GalNAcα1-3(Fucα1-2)Galβ1-3GalNAcβ1-3Gal	Blood group A antigen pentaose type 4	894.82
20C	Galα1-3(Fucα1-2)Galβ1-3GalNAcβ1-3Gal	Blood group B antigen pentasaccaride type 4	853.76
	**Sialylated glycans**		
10A	Neu5Acα2-3Galβ1-3(Fucα1-4)Glc*N*Ac	Sialyl Lewis^{a} (S Lea)	820.74
10B	Neu5Acα2-3Galβ1-4(Fucα1-3)Glc*N*Ac	Sialyl Lewis^{x} (S Lex)	820.74
10C	Neu5Acα2-3Galβ1-3Glc*N*Acβ1-3Galβ1-4Glc	Sialyllacto-N-tetraose a	
10D	Galβ1-4(Fucα1-3)Glc*N*Acβ1-6(Neu5Acα2-6Galβ1-4Glc*N*Acβ1-3)Galβ1-4Glc	Monosialyl, monofucosyllacto-N-neohexose	1510.36
10E	Neu5Acα2-3Galβ1-3(Neu5Acα2-6)GalNAc	Disialyl-TF	965.86
10H	Neu5Acα2-6Galβ1-3GlcNAcβ1-3Galβ1-4(Fucα1-3)Glc	Sialyllacto-N-fucopentaose VI	1145.03
10I	Galβ1-3GlcNAcβ1-3(Neu5Acα2-6Galβ1-4GlcNAcβ1-6)Galβ1-4Glc	Monosialyllacto-N-hexaose	1364.22
10J	Neu5Acα2-6Galβ1-3GlcNAcβ1-3(Galβ1-4GlcNAcβ1-6)Galβ1-4Glc	Monosialyllacto-N-neohexaose	1364.22
10K	Neu5Acα2-3Galβ1-4Glc*N*Ac	3'-Sialyllactosamine	674.6
10L	Neu5Acα2-6Galβ1-4Glc*N*Ac	6'-Sialyllactosamine	674.6
10M	Neu5Acα2-3Galβ1-3GlcNAcβ1-3Galβ1-4Glc	LS-Tetrasaccharide a (LSTa)	998.88
10N	Galβ1-3(Neu5Acα2-6)Glc*N*Acβ1-3Galβ1-4Glc	LS-Tetrasaccharide b (LSTb)	998.88
10O	Neu5Acα2-6Galβ1-4Glc*N*Acβ1-3Galβ1-4Glc	LS-Tetrasaccharide c (LSTc)	998.88

10P	Neu5Acα2-3Galβ1-3(Neu5Acα2-6)Glc*N*Acβ1-3Galβ1-4Glc	Disialyllacto-N-tetraose	1290.14
11A	Neu5Acα2-3Galβ1-4Glc	3'-Sialyllactose	633.55
11B	Neu5Acα2-6Galβ1-4Glc	6'-Sialyllactose	633.55
11C	(Neu5Acα2-8Neu5Ac)n (n<50)	Colominic acid	
18A	Neu5Acα2-3Galβ1-4GlcNAcβ1-3Galβ1-4Glc	LS-Tetrasaccharide d	1020.86
18K	9-NAc-Neu5Ac	9-acetamido-9-deoxy-N-acetyl-a-D-neurminic acid	351.31
18O	Neu5Gc	N-glycolylneuraminic acid	325.27
19K	Neu5Acα2-3Galβ1-4(Fucα1-3)GlcNAcβ1-3Gal	Sialyl lewis X pentaose	1004.86
	**Glycosaminoglycans - high and low molecular weight**		
12A	Neocarratetraose-41, 3-di-O-sulphate (Na+)	$C_{24}H_{36}O_{25}S_2Na_2$ (Mixed anomers. Tetrasaccharide of regular κ - carrageenan)	834.64
12B	Neocarratetraose-41-O-sulphate (Na+)	$C_{24}H_{37}O_{22}SNa$ (Mixed anomers. Derived from C1003 by removal of the non-reducing terminal 4-sulphate)	732.59
12C	Neocarrahexaose-24,41, 3, 5-tetra-O-sulphate (Na+)	$C_{36}H_{52}O_{40}S_4Na_4$ (Mixed anomers. A hybrid sequence comprising carrageenan disaccharides in the order k-i-k, derived from the carrageenan from *Chondrus crispus*)	1344.99
12D	Neocarrahexaose-41, 3, 5-tri-O-sulphate (Na+)	$C_{36}H_{53}O_{37}S_3Na_3$ (Mixed anomers. Hexasaccharide of regular κ-carrageenan)	1242.95
12E	Neocarraoctaose-41, 3, 5, 7-tetra-O-sulphate (Na+)	$C_{48}H_{70}O_{49}S_4Na_4$ (Mixed anomers. Octasaccharide of regular κ-carrageenan)	1651.26
12F	Neocarradecaose-41, 3, 5, 7, 9-penta-O-sulphate (Na+)	$C_{60}H_{87}O_{61}S_5Na_5$ (Mixed anomers. Decasaccharide of regular κ-carrageenan)	2059.57
12G	ΔUA-2S-GlcNS-6S	$C_{12}H_{15}NO_{19}S_3Na_4$ (Predominant disaccharide produced from heparin by heparinase I and II)	665.4
12H	ΔUA-GlucNS-6S	$C_{12}H_{16}NO_{16}S_2Na_3$ (Produced from heparinase II digestion of heparin and heparin sulphate)	563.35
12I	ΔUA-2S-GlucNS	$C_{12}H_{16}NO_{16}S_2Na_3$ (Produced from heparin by digestion with heparinase I and II)	563.35

12J	ΔUA-2S-GlcNAc-6S	$C_{14}H_{18}NO_{17}S_2Na_3$ (Minor component produced from heparin by heparinase II)	605.39
12K	ΔUA-GlcNAc-6S	$C_{14}H_{19}NO_{14}SNa_2$ (Product of the action of heparinases II and III on heparin and heparan sulphate)	503.34
12L	ΔUA-2S-GlcNAc	$C_{14}H_{19}NO_{14}SNa_2$ (Minor product of the action of heparinase II on heparin)	503.34
12M	ΔUA-GlcNAc	$C_{14}H_{20}NO_{11}Na$ (Produced from heparin sulphate by digestion With heparinase III)	401.3
12N	ΔUA-GalNAc-4S (Delta Di-4S)	$C_{14}H_{19}NO_{14}SNa_2$ (Produced from various chondroitin sulphates By the action of chondroitinases ABC, B and AC-1)	503.34
12O	ΔUA-GalNAc-6S (Delta Di-6S)	$C_{14}H_{19}NO_{14}SNa_2$ (Produced from various chondroitin sulphates By the action of chondroitinases ABC, AC-1 and C)	503.34
12P	ΔUA-GalNAc-4S,6S (Delta Di-disE)	$C_{14}H_{18}NO_{17}S_2Na_3$ (Produced from various chondroitin sulphates By the action of chondroitinases ABC, B and AC-1)	605.39
13A	ΔUA-2S-GalNAc-4S (Delta Di-disB)	$C_{14}H_{18}NO_{17}S_2Na_3$ (Produced from various chondroitin sulphates by action of chondroitinase ABC and/or B. Most typically from chondroitin sulphate B (dermatan sulphate))	605.39
13B	ΔUA-2S-GalNAc-6S (Delta Di-disD)	$C_{14}H_{18}NO_{17}S_2Na_3$ (Produced from various chondroitin sulphates by the action of chondroitinase ABC)	605.39
13C	ΔUA-2S-GalNAc-4S-6S (Delta Di-tisS)	$C_{14}H_{17}NO_{20}S_3Na_4$ (Produced as a minor component by the action of chondroitinase ABC on various chondroitin sulphates, particularly B)	707.43
13D	ΔUA-2S-GalNAc-6S (Delta Di-UA2S)	$C_{14}H_{19}NO_{14}SNa_2$ (Produced as a minor component from various chondroitin sulphates by the action of chondroitinase ABC)	503.34
13E	ΔUA-GlcNAc (Delta Di-HA)	$C_{14}H_{20}NO_{11}Na$ (The only unsaturated disaccharide produced from hyaluronic acid by the action of chondroitinase ABC or AC-1)	401.3

13F	(GlcAβ1-3Glc*N*Acβ1-4)n (n=4)	Hyaluronan fragments (4mer)	
13G	(GlcAβ1-3Glc*N*Acβ1-4)n (n=8)	Hyaluronan fragment (8mer)	
13H	(GlcAβ1-3Glc*N*Acβ1-4)n (n=10)	Hyaluronan fragment (10mer)	
13I	(GlcAβ1-3Glc*N*Acβ1-4)n (n=12)	Hyaluronan fragment (12mer)	
13J	(GlcA/IdoAα/β1-4Glc*N*Acα1-4)n (n=200)	Heparin	
13K	(GlcA/IdoAβ1-3(±4/6S)Gal*N*Acβ1-4)n (n<250)	Chondroitin sulfate	
13L	((±2S)GlcA/IdoAα/b1-3(±4S)Gal*N*Acβ1-4)n (n<250)	Dermatan sulfate	
13M	(GlcA/IdoAβ1-3(±6S)Gal*N*Acβ1-4)n (n<250)	Chondroitin 6-sulfate	
13N	HA - 4 10mM		775
130	HA - 6 10mM		1155.6
13P	HA - 8 9.7mM		1534.7
14A	HA 10 7.83mM		1913.8
14B	HA-12 6.5mM		2293.4
14C	HA-14 5.6mM		2672.5

14D	HA-16 4.9mM		
14E	HA 30000 da 2.5mg/ml		
14F	HA 107000 da 2.5mg/ml		
14G	HA 190000 da 2.5 mg/ml		
14H	HA 220000 da 2.5 mg/ml		
14I	HA 1600000 da 2.5 mg/ml		
14J	Heparin sulfate 5 mg/ml		
14K	β1-3Glucan		
14L	Chondroitin disaccharide Δdi-OS, sodium salt	$C_{14}H_{20}NNaO_{11}$ (produced from various chondriotin sulfates by the action of chondriotinases ABC, AC-1 and C)	401.3
14M	ΔUA→2S-GlcN-6S	Heparin unsaturated disaccharide I-H, the major component from the action of heparinase II on de-N-sulfated heparin	541.37
14N	ΔUA→GlcN-6S	Heparin unsaturated disaccharide II-H, the major component from the action of heparinase II on de-N-sulfated heparin	439.32
14O	ΔUA→2S-GlcN	Heparin unsaturated disaccharide III-H, the major component from the action of heparinase II on de-N-sulfated heparin	439.32
14P	ΔUA→GlcN	Heparin unsaturated disaccharide iV-H is obtained from the action of heparinases on heparin	337.28
18I	GlcA	D-Glucuronic acid	194.14
	**Terminal Glucose structures**		
18J	6-O-(H_2PO_4)-Glc	D-Glucose-6-phosphate	260.14
19O	Glcα1-4Glcα1-4Glc	Maltotriose	504.44
19P	Glcα1-4Glcα1-4Glcα1-4Glc	Maltotetraose	666.58
	**Ganglioside structures**		

17A	GalNAcβ1-4Galβ1-4Glc	asialo GM2	545.48
17B	Galβ1-3GalNAcβ1-4Galβ1-4Glc	asialo GM1	707.62
17C	Galβ1-3GalNAcβ1-4(Neu5Acα2-8Neu5Acα2-8 Neu5Acα2-3)Galβ1-4Glc	GT1c ganglioside sugar	1647.33
17D	Neu5Acα2-8Neu5Acα2-3Galβ1-3GalNAcβ1-4(Neu5Acα2-3)Galβ1-4Glc	GT1a ganglioside sugar	1647.33
17E	Galβ1-3GalNAcβ1-4(Neu5Acα2-8Neu5Acα2-3)Galβ1-4Glc	GD1b ganglioside sugar	1334.09
17F	Neu5Acα2-3Galβ1-3GalNAcβ1-4(Neu5Acα2-3)Galβ1-4Glc	GD1a Ganglioside sugar	1334.09
17G	Neu5Acα2-3Galβ1-3GalNAcβ1-4Galβ1-4Glc	GM1b ganglioside sugar	1020.86
17H	Galβ1-3GalNAcβ1-4(Neu5Acα2-3)Galβ1-4Glc	GM1a ganglioside sugar	1020.86
17I	Fucα1-2Galβ1-3GalNAcβ1-4(Neu5Acα2-3)Galβ1-4Glc	fucosyl GM1 ganglioside sugar	1168
17J	GalNAcβ1-4(Neu5Acα2-8Neu5Acα2-8Neu5Acα2-3)Galβ1-4Glc	GT2 ganglioside sugar	1485.19
17K	GalNAcβ1-4(Neu5Acα2-8Neu5Acα2-3)Galβ1-4Glc	GD2 gangliosifde sugar	1171.95
17L	GalNAcβ1-4(Neu5Acα2-3)Galβ1-4Glc	GM2 ganglioside sugar	858.72
17M	Neu5Acα2-8Neu5Acβ2-8Neu5Acα2-3Galβ1-4Glc	GT3 ganglioside sugar	1282
17N	Neu5Acα2-8Neu5Acα2-3Galβ1-4Glc	GD3 ganglioside sugar	968.76
17O	Neu5Acα2-3Galβ1-4Glc	GM3 ganglioside sugar	655.53
17P	Neu5Acα2-3Galβ1-3GalNAcβ1-4(Neu5Acα2-8Neu5Acα2-3)Galβ1-4Glc	GT1b ganglioside sugar	
	**Complex type N-glycans**		
19A	Galβ1-4GlcNAcβ1-2Manα1-3(Galβ1-4GlcNAcβ1-2Manα1-6Man)β1-4GlcNAcβ1-4(Fucα1-6)GlcNAc	Asialo galactosylated, fucosylated biantennary	1787.63
19B	Galβ1-4GlcNAcβ1-2(Galβ1-4GlcNAcβ1-4)Manα1-3(Galβ1-4GlcNAcβ1-2(Galβ1-4GlcNAcβ1-6)Manα1-6Man)β1-4GlcNAcβ1-4GlcNAc	Asialo, galactosylated, tetranatennary, N-linked glycan	2372.15

19C	Neu5Acα2-6Galβ1-4GlcNAcβ1-2Manα1-3(Galβ1-4GlcNAcβ1-2Manα1-6)Manβ1-4GlcNAcβ1-4GlcNAc	Monosialo(2,6), biantennary (A1)	1932.74
19D	Neu5Acα2-6Galβ1-4GlcNAcβ1-2Manα1-3(Neu5Acα2-6Galβ1-4GlcNAcβ1-2Manα1-6)Manβ1-4GlcNAcβ1-4GlcNAc	Disialo (2,6) biantennary (A2)	2224
19E	Galβ1-4GlcNAcβ1-2Manα1-3(Galβ1-4GlcNAcβ1-2Manα1-6)Manβ1-4GlcNAcβ1-4GlcNAc	Asialo, galactoyslated, biantennary (NA2)	1641.49
19F	Neu5Acα2-6Galβ1-4GlcNAcβ1-2Manα1-3(Neu5Acα2-6Galβ1-4GlcNAcβ1-2Manα1-6)Manβ1-4GlcNAcβ1-4(Fucα1-6)GlcNAc	Disialo, galactosylated, fucosylated, biantennary (A2F)	2370.14
19G	Neu5Acα2-6Galβ1-4GlcNAcβ1-2(Neu5Acα2-6Galβ1-4GlcNAcβ1-4)Manα1-3(Neu5Acα2-6Galβ1-4GlcNAcβ1-2Manα1-6)Manβ1-4GlcNAcβ1-4GlcNAc	Trisialylated, galactosylated, triantennary (A3)	2880.59
19H	GlcNAcβ1-2(GlcNAcβ1-4)Manα1-3(GlcNAcβ1-2Manα1-6)GlcNAcβ1-4Manβ1-4GlcNAcβ1-4GlcNAc	Asialo, agalacto, bisected triannary (NGA3B)	1723.59

Supplementary Table 2. A list of all glycans printed on the microarray

**Strain**	**Relevant features**	**Source**
BW25113	*Δ(araD-araB)567 ΔlacZ4787*(::rrnB-3) λ^- *rph-1 Δ(rhaD-rhaB)568 hsdR514*	Keio collection
BW25113*ΔytfB*	BW25113 *ΔytfB::frt*	This study
BW25113 *ytfB-FLAG*	BW25113 *ytfB::ytfB-FLAG::frt*	This study
UTI89	O18:K1:H7	[1]
UTI89 *ΔytfB*	UTI89 *ΔytfB::frt*	This study
UTI89 *ytfB::kan*	UTI89 *ytfB::kan* (Kan^R)	This study
UTI89 pGEN	UTI89/pGEN-MCS (Amp^R)	This study
UTI89 *ΔytfB* pGEN	UTI89 *ΔytfB::frt*/pGEN-MCS (Amp^R)	This study
UTI89 *ΔytfB* pGEN-ytfB	UTI89 *ΔytfB::frt*/pGEN-ytfB (Amp^R)	This study
**Plasmid**	**Description**	**Source**
pGEN-ytfB	pGEN-MCS with the *ytfB* ORF sequence, (1-639), and promoter region, (100 nucleotides upstream of ORF), with stop codon included.	This study
pETMCSIII-ytfB	pETMCSIII with the extracellular domain of *ytfB* (52-212)	This study

Supplementary Table 3. E. coli strains and plasmids used in this study

Name	Sequence	description
ALB264	GCT TAT TCA TAG GCT ATG ATT GAG GAA CAA GAC GCG GAG CAG GAG GAA ACC GTG TAG GCT GGA GCT GCT TCG AAG	Forward primer for deletion of *ytfB* using lambda Red recombination
ALB265	TCA TCA AAG AGA CAA TCC TTA GCT GGC TTT TGG TTG ACC CTT CAT CTA ATT CCG GGG ATC CGT CGA CCT GCA G	Reverse primer for deletion of *ytfB* using lambda Red recombination
ALB268	GC CAG CCG GAT GGC AGT TTT ATT CGT GCG CGG GAC TAC AAG *GAC GAT GAC GAC AAA TAG* GTG TAG GCT GGA GCT GCT TCG AAG	forward primer for constructing *ytfB* with C-terminal FLAG by lambda Red recombination
ALB325	AAA AAA **CAT ATG** GAT ACG CCC AAC GCG CCG	forward primer for cloning extracellular domain of *ytfB* into pETMCSIII
ALB326	AAA AAA **CCA TGG** CTA CCG CGC ACG AAT AAA ACT GCC ATC C	reverse primer for cloning *ytfB* into pETMCSIII

EP1	AAA TTT **GAA TTC** CTA CCG CGC GCG AAT AAA ACT	Forward primer for cloning UTI89 *ytfB* into pGEN-MCS
EP2	AAA TTT **GGA TCC** CCC GTA TGA ATA TAA TAA AGG CGG GTT TAC	Reverse primer for cloning UTI89 *ytfB* into pGEN-MCS

Supplementary Table 4. Primers used in this study.

Restriction enzymes are bold, synthetic FLAG is in italics and FRT-flanked sequences are underlined

Supplemental Experimental procedures

LPS extraction. Lipopolysaccharide (LPS) was extracted from 5 ml overnight culture of UTI89 and Δ*ytfB* using a LPS extraction kit (iNTrON Biotechnology) following manufacturer's instructions. Equal amounts of LPS were then separated on a pre-cast 4-12% SDS-polyacrylamide gradient gel (Novex, Thermo Fischer Scientific) and silver-stained using the silver stain plus kit (BioRad).

Lysozyme sensitivity assay. For solid media assays, UTI89 and Δ*ytfB* were grown in LB overnight. The following day, the optical density of cultures was normalised, and 250 µl of culture was added to 5 ml of LB top agar (0.7% agar). This was poured over the top of LB plates and allowed to set. Ten microliters of 50 mg/ml lysozyme was spotted on top of the centre of the agar. Plates were incubated overnight at 30 °C and the diameter of the zones of lysis were measured. For liquid lysozyme assays, overnight cultures of UTI89 and Δ*ytfB* were washed in PBS before being added to the wells of a 96-well plate to a final OD_{600} of 0.4. Lysozyme was added to a final concentration of 10 mg/ml and plates were incubated at 37 °C with shaking. Absorbance (OD_{600}) was recorded every 10 min and the reduction of absorbance, indicating lysis, was calculated as a fraction of the starting optical density.

Growth conditions. Bacteria were cultured in synthetic human urine[2] or M9 + glucose minimal media at 37 °C with shaking. Growth in minimal medium was supplemented with 1 mg/ml nicotinamide to account for UTI89 auxotrophy[3, 4]. Human urine was collected from a male donor and filter sterilised. Concentrated urine was classified as urine that had a specific gravity of 1.02 g/ml or higher. Growth in fetal bovine serum (FBS) was done in M9 minimal medium containing 10% FBS. Samples were collected at mid-exponential growth phase and fixed with 2.4% formaldehyde. Cells were then mounted on poly-L-lysine coasted slides and viewed by phase contrast using a Zeiss Axioplan 2 fluorescence microscope equipped with a Plan ApoChromat (100x NA 1.4; Zeiss) objective lens and an AxioCam MRm cooled charged-coupled-device (CCD) camera. Cell length values were quantified using AxioVision software v4.6 (Zeiss) with default measurement settings.

Western blot. UTI89 and UTI89Δ*ytfB* were grown overnight in LB medium statically to promote fimbrial expression. Whole cell lysates were prepared and separated on a 16 % SDS-PAGE gel. Western blots were performed as using standard procedure and probed with either α-fim or α-FtsZ antibodies at 1:500 or 1:10 000, respectively.

Static infection of bladder cells. The human bladder cell line PD07i was cultured to confluence in 24-well cell culture plates at 37 ˚C in 5% CO_2 in EpiLife + HKGS. Bacterial strains were grown statically overnight at 37 °C in 5ml LB medium to promote fimbrial expression. Bacterial cultures were diluted to 10^9 cells/ml (OD_{600}=1). To infect PD07i cells, 0.1 µl/mm^2 of bacterial culture was added to each well,

to give an MOI of 100. Cell culture plates were centrifuged for 5 min, 600 *g* at 24°C to force the bacteria onto the cell surface then incubated at 37°C with 5% CO_2 for 2 hours. For the adhesion assay, cells were washed with PBS 6 times. To lyse the mammalian cells, 1ml of lysis solution (0.5 % Trypsin-EDTA, 0.1% Triton X-100) was added and incubated for 15 minutes at 37 ˚C in 5% CO_2, and bacterial cells were resuspended by pipetting. Dilutions of the bacteria were plated onto LB agar plates to count the CFU/ml. For bacterial internalisation and intracellular growth assays, the EpiLife media was removed and replace with 4 µl/mm^2 of EpiLife + HKGS + 100 µg/ml gentamycin. This was incubated statically for 1 h (internalisation) or 24 h (intracellular growth). The media was then removed and the wells washed with PBS four times. The bladder cells were lysed and plated as for the adhesion assay. Averages of technical replicates were calculated and the data was normalised to the wild -type control before means and standard errors of the means were calculated for the biological replicates. Unpaired student T-tests were then performed to test for significant differences between strains.

Murine CAUTI and UTI model. The urinary tract infection model was carried out as previously described [5]. Briefly, bacterial strains were grown in 15 mL LB media for 16-18 hrs with shaking at 37°C. Cells were pelleted, resuspended in 5 mL sterile 1× PBS and the OD was normalized to 2×10^8 CFU/mL. For competitive CAUTI, equal volume of each strain was mixed prior to infection. Firstly, groups of 5 isofluorane-anesthetized female wild-type C57BL/6 mice (7-8 weeks old, 22 to 25g; InVivos, Singapore) were infected via intraurethral catheterization (polyethylene catheter, 5mm long, 0.61mm) with 50 µL of bacteria inoculum. Mice were euthanized by carbon dioxide inhalation and cervical dislocation 24 hpi after transurethral challenge, and bladder, kidney pairs were aseptically excised, weighed and homogenized in 1 mL and 0.8 mL 1× PBS, respectively using a homogenizer (Pro200, SPD scientific, Singapore) for approximately 10 sec at high speed. The catheter implant was also retrieved from each bladder and placed in 1mL 1× PBS prior to water bath sonication at r.t.p for 5 mins. Serial homogenate dilutions were plated onto MacConkey agar selection plate with and without kanamycin for CFU enumeration. The limit of detection is 40 CFU. The murine CAUTI model was performed twice independently and murine that lose the catheter at the time of sacrifice was omitted and a P value of less than 0.05 was considered significant.

For competitive UTI, the mice were infected with 50 µL of 2×10^8 CFU/mL of mixed bacterial inoculum and the steps for euthanization, excision of bladder and kidneys as well as the preparation of the homogenate is the same as above.

To measure the fitness of the strains in causing infection, we calculate the competitive indices (CI) using the formula below:

$$\mathrm{CI\ (UTI89)} = \left(\frac{\mathrm{UTI89(output)}}{\mathrm{ytfB :: kan(output)}}\right) / \left(\frac{\mathrm{UTI89(input)}}{\mathrm{ytfB :: kan(input)}}\right)$$

$$\mathrm{CI}\ (\Delta ytfB) = \left(\frac{\Delta ytfB\mathrm{(output)}}{\mathrm{ytfB :: kan(output)}}\right) / \left(\frac{\Delta ytfB\mathrm{(input)}}{\mathrm{ytfB :: kan(input)}}\right)$$

The input indicates the initial inoculum used to infect the murine wound. The output indicates the inoculum recovered from the bladders, kidneys or the implants 24 hpi.

Supplemental References

1. Mulvey, M.A., Schilling, J.D. & Hultgren, S.J. Establishment of a persistent Escherichia coli reservoir during the acute phase of a bladder infection. *Infect Immun* **69**, 4572-9 (2001).
2. Ipe, D.S., Horton, E. & Ulett, G.C. The Basics of Bacteriuria: Strategies of Microbes for Persistence in Urine. *Front Cell Infect Microbiol* **6**, 14 (2016).
3. Bahrani-Mougeot, F.K. et al. Type 1 fimbriae and extracellular polysaccharides are preeminent uropathogenic Escherichia coli virulence determinants in the murine urinary tract. *Mol Microbiol* **45**, 1079-93 (2002).
4. Li, Z., Bouckaert, J., Deboeck, F., De Greve, H. & Hernalsteens, J.P. Nicotinamide dependence of uropathogenic Escherichia coli UTI89 and application of nadB as a neutral insertion site. *Microbiology* **158**, 736-45 (2012).
5. Hung, C.S., Dodson, K.W. & Hultgren, S.J. A murine model of urinary tract infection. *Nat Protoc* **4**, 1230-43 (2009).
